# The Associations of Maternal Health Characteristics, Newborn Metabolite Concentrations, and Child Body Mass Index among US Children in the ECHO Program

**DOI:** 10.3390/metabo13040510

**Published:** 2023-04-01

**Authors:** Brittney M. Snyder, Tebeb Gebretsadik, Nina B. Rohrig, Pingsheng Wu, William D. Dupont, Dana M. Dabelea, Rebecca C. Fry, Susan V. Lynch, Cindy T. McEvoy, Nigel S. Paneth, Kelli K. Ryckman, James E. Gern, Tina V. Hartert, on behalf of Program Collaborators for Environmental Influences on Child Health Outcomes

**Affiliations:** 1 Department of Medicine, Vanderbilt University Medical Center, Nashville, TN 37203, USA; 2 Department of Biostatistics, Vanderbilt University Medical Center, Nashville, TN 37203, USA; 3 Lifecourse Epidemiology of Adiposity and Diabetes (LEAD) Center, University of Colorado Anschutz Medical Campus, Aurora, CO 80045, USA; 4 Department of Environmental Sciences and Engineering, Gillings School of Public Health, University of North Carolina at Chapel Hill, Chapel Hill, NC 27599, USA; 5 Department of Medicine, University of California, San Francisco, CA 94143, USA; 6 Department of Pediatrics, Oregon Health and Science University, Portland, OR 97239, USA; 7 Department of Epidemiology and Biostatistics, College of Human Medicine, Michigan State University, East Lansing, MI 48824, USA; 8 Department of Pediatrics and Human Development, College of Human Medicine, Michigan State University, East Lansing, MI 48912, USA; 9 Department of Epidemiology and Biostatistics, Indiana University School of Public Health—Bloomington, Bloomington, IN 47405, USA; 10 Department of Pediatrics, University of Wisconsin School of Medicine and Public Health, Madison, WI 53792, USA; 11 Department of Pediatrics, Vanderbilt University Medical Center, Nashville, TN 37203, USA

**Keywords:** prenatal and perinatal exposures, maternal stressors, fetal metabolic programming, newborn metabolites, child growth patterns

## Abstract

We aimed first to assess associations between maternal health characteristics and newborn metabolite concentrations and second to assess associations between metabolites associated with maternal health characteristics and child body mass index (BMI). This study included 3492 infants enrolled in three birth cohorts with linked newborn screening metabolic data. Maternal health characteristics were ascertained from questionnaires, birth certificates, and medical records. Child BMI was ascertained from medical records and study visits. We used multivariate analysis of variance, followed by multivariable linear/proportional odds regression, to determine maternal health characteristic-newborn metabolite associations. Significant associations were found in discovery and replication cohorts of higher pre-pregnancy BMI with increased C0 and higher maternal age at delivery with increased C2 (C0: discovery: aβ 0.05 [95% CI 0.03, 0.07]; replication: aβ 0.04 [95% CI 0.006, 0.06]; C2: discovery: aβ 0.04 [95% CI 0.003, 0.08]; replication: aβ 0.04 [95% CI 0.02, 0.07]). Social Vulnerability Index, insurance, and residence were also associated with metabolite concentrations in a discovery cohort. Associations between metabolites associated with maternal health characteristics and child BMI were modified from 1–3 years (interaction: *p* < 0.05). These findings may provide insights on potential biologic pathways through which maternal health characteristics may impact fetal metabolic programming and child growth patterns.

## 1. Introduction

The developing fetus is vulnerable to maternal exposures, including psychosocial factors, lifestyle factors, external environmental exposures, and biological factors (e.g., inflammation, gut microflora, etc.), and can adapt in response to these exposures [1]. This process of adaptation, sometimes described as fetal programming, is important in normal development and can increase the chance of survival in early life [2,3]. However, some maternal exposures can detrimentally alter fetal programming, predisposing the infant to later life disease development [2]. Changes in fetal and neonatal cellular response is one plausible mechanism through which maternal exposures can impact fetal programming and lead to subsequent disease development in the offspring [2]. While research has primarily focused on fetal epigenetic modifications [4], downstream neonatal metabolic alterations may constitute a biomarker reflective of the biologic effect of risk factors and may play a mediating role in the observed associations between in utero stressors and later life disease development.

Metabolites are end products of cellular regulatory processes and measures of genetically influenced responses to exposure [5,6]. Metabolism provides the body with energy used for growth, development, movement, and reproduction [7]. While many types of metabolites exist [8], levels of certain metabolites directly involved in vital processes—such as free carnitine, acylcarnitines, and amino acids—are tightly regulated [9,10]. Thus, perturbations in concentrations of these metabolites may be indicative of metabolic pathways involved in disease pathogenesis [9]. Assessing the influence of maternal health characteristics on newborn concentrations of free carnitine, acylcarnitines, and amino acids could provide important insights on fetal metabolic programming and pathways underlying later life metabolic dysfunction and disease.

**Figure 1:**
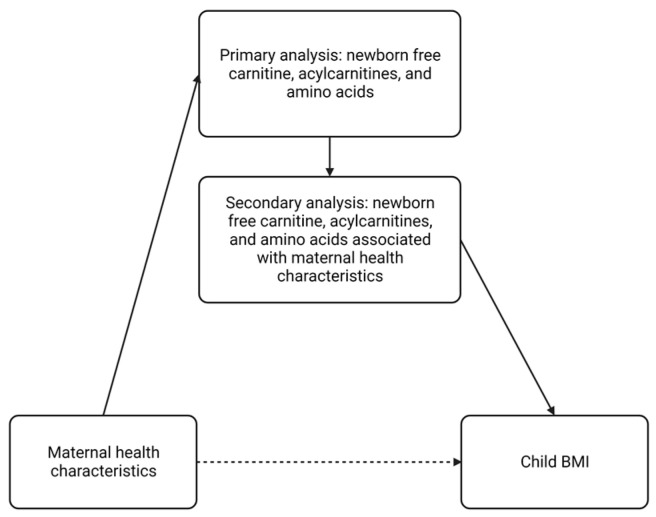
Directed acyclic graph of study objectives. BMI—body mass index. This figure was created with BioRender.com (accessed on 23 February 2023).

We hypothesized that: (1) maternal health characteristics are associated with newborn free carnitine, acylcarnitines, and amino acid concentrations and (2) metabolites associated with maternal health characteristics are also associated with subsequent childhood growth patterns. The study objectives were first to evaluate the impact of maternal health characteristics on newborn free carnitine, acylcarnitine, and amino acid concentrations in a multi-cohort study using targeted blood metabolic data from newborn screening (NBS) programs. Next, we assessed associations between newborn metabolites associated with maternal health characteristics and child body mass index (BMI) from ages 1–3 years (Figure 1). This study is a first step towards assessing biologic pathways through which maternal health characteristics may impact fetal metabolic programming and child growth patterns.

## 2. Materials and Methods

### 2.1. Study Design and Populations

This multi-site study included three birth cohorts from the National Institutes of Health (NIH) Environmental Influences on Child Health Outcomes (ECHO) Program (https://echochildren.org/ (accessed on 4 January 2023)). The cohorts included in the present study (INSPIRE, MARCH, and Healthy Start) have been described previously [11,12]. We linked NBS blood metabolic data with each of these cohorts and included enrolled infants with linked NBS blood metabolic data. The protocol and informed consent documents were approved by the Vanderbilt University Medical Center, Michigan State University, University of Colorado, Tennessee Department of Health, Michigan Department of Health and Human Services, and Colorado Department of Public Health and Environment Institutional Review Boards.

### 2.2. Newborn Screening Metabolic Data Collection

NBS metabolic data include targeted measurement of free carnitine, acylcarnitines, and amino acids. Collection of blood spot cards for NBS is standardized, requiring collection by a health care professional within 24–48 h after birth and sent to the respective state laboratory for routine testing [13]. Tandem mass spectrometry (MS/MS) was then used to quantitatively measure metabolite concentrations using the calculated ratio of the signal from each metabolite to the signal from the known amount of internal standard [14]. Quantified results were stored on state public health department servers.

Existing NBS metabolic data were provided for infants enrolled in the cohorts by the NBS programs at the Tennessee Department of Health, Michigan Department of Health and Human Services, and Colorado Department of Public Health and Environment. Metabolites measured in each cohort are listed in Table S1. Data were provided for infants who did not screen positive for any inherited disorder (i.e., metabolite concentrations were within the normal range, representing >99% of infants in the US [15]) to reduce the risk of potential participant identification and remove skewed metabolic profiles due to inborn errors of metabolism. We then linked the metabolic data with demographic and clinical data from each of the cohorts.

### 2.3. Maternal Health Characteristics, Child BMI, and Covariate Ascertainment

We assessed several maternal health characteristics based on cohort availability. Maternal health characteristics (e.g., prenatal smoking, pre-pregnancy BMI, education, occupational status, marital status, age at delivery, asthma, gestational diabetes, and mode of delivery) were ascertained from questionnaires administered at enrollment for INSPIRE participants, birth certificates and questionnaires administered during pregnancy for MARCH participants, and medical records at delivery and questionnaires administered during pregnancy for Healthy Start participants (Table S2).

We ascertained child BMI at ages 1, 2, and 3 years in subsets of the INSPIRE and Healthy Start cohorts with available weight and height measurements. Child BMI was ascertained through medical record abstraction for participants in the Healthy Start cohort and from medical records and/or study visits for participants in the INSPIRE cohort. Weight and height measurements were collected on the same date, within each year, for Healthy Start children. We calculated estimated recumbent length/standing height at weight measurement date for INSPIRE children with lengths/heights and weights measured on different days within each year (year 1: *n* = 60, year 2: *n* = 186, year 3: *n* = 17) using World Health Organization (WHO) growth charts [16] for children age < 2 years (recumbent length) and Centers for Disease Control (CDC) growth charts [17] for children age 2–3 years (standing height) [18].

Covariates (e.g., birth weight [grams], gestational age [weeks], infant race, infant ethnicity, and sex) were collected from enrollment questionnaires for INSPIRE participants. Birth weight, gestational age, and sex were ascertained from birth certificates for MARCH participants, while infant race and ethnicity were collected from questionnaires administered at infant age 3 months. For Healthy Start participants, sex was ascertained from delivery questionnaires. Infant race and ethnicity were ascertained from questionnaires administered at infant age 6 months. Birth weight was derived from several sources using the following hierarchy: (1) newborn medical record abstraction, (2) newborn physical exam performed within a week after birth, (3) self-reported at delivery interview, (4) self-reported at infant 6-month visit, and (5) self-reported at infant 18-month visit. Gestational age was ascertained from medical records for 96% of participants and delivery questionnaires for 3% of participants (1% of participants were missing information on gestational age).

### 2.4. Statistical Analysis

We compared maternal characteristics, infant characteristics, and metabolite concentrations between the cohorts using Kruskal–Wallis or Pearson χ^2^ test, as appropriate. We used multiple imputation (n = 5 iterations) using Fully Conditional Specification (FCS) implemented by the Multivariate Imputation by Chained Equations (MICE) algorithm for each cohort separately to estimate possible values for missing data [19]. All analyses were performed using multiply imputed datasets. For the primary analysis, we pooled the cohorts with the largest and smallest number of infants with linked NBS metabolic data in a discovery phase (INSPIRE and MARCH), and we utilized the Healthy Start cohort in a replication phase to have similar regression power. Metabolites measured in both the INSPIRE and MARCH cohorts were included in the analysis (*n* = 31, Table S1). NBS metabolite concentrations for the study populations are shown in Table S3.

**Figure 2:**
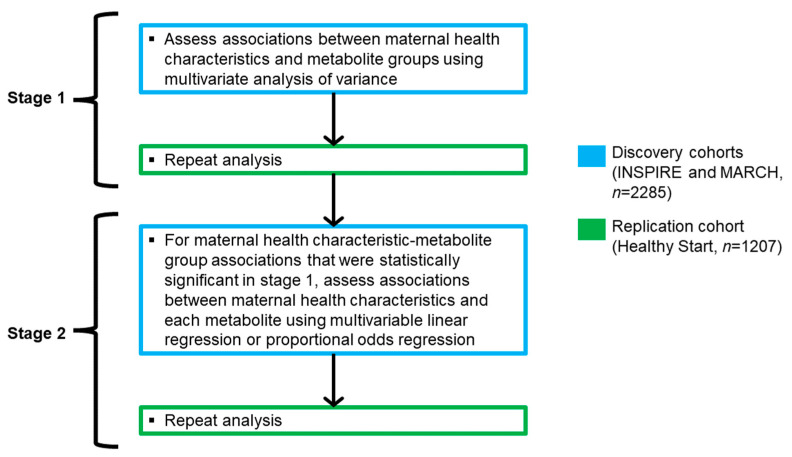
Diagram of *a priori* statistical plan. Metabolites that did not fit into one of the pre-specified metabolite groups (*n* = 6) were also assessed in stage 2.

Our *a priori* statistical plan consisted of a two-stage process (Figure 2). In stage one, we assessed the associations between maternal health characteristics and established metabolite groups [20] (short-, medium-, and long-chain acylcarnitines and amino acids [Table S4]) in the discovery cohorts using multivariate analysis of variance (MANOVA), adjusting for cohort, birth weight, gestational age, infant race, infant ethnicity, and sex. Assessing global associations between maternal health characteristics and metabolite groups through MANOVA, compared to each metabolite separately, helped reduce the multiple testing burden. As MANOVA assumes interval measurement of dependent variables, only metabolites with continuous distributions (*n* = 26) were included in these pre-specified groups (Figure S1). We then repeated this analysis in the replication cohort, adjusting for the same covariates (excluding cohort).

In stage two, for maternal health characteristic-metabolite group associations that were statistically significant in both the discovery and replication cohorts, we assessed the relationships between maternal health characteristics and each metabolite, including free carnitine (C0) which did not fit into one of the pre-specified metabolite groups, using multivariable linear regression. We also assessed the relationships between maternal health characteristics and *n* = 5 metabolites with ordinal distributions (tiglylcarnitine [C5:1], decadienoylcarnitine [C10:2], 3-hydroxytetradecanoylcarnitine [C14-OH], 3-hydroxypalmitoylcarnitine [C16-OH], and 3-hydroxyoleoylcarnitine [C18:1-OH] [Figure S1]), which did not meet MANOVA assumptions, using proportional odds regression. For maternal health characteristic-metabolite associations that remained statistically significant in the discovery cohorts, we repeated this analysis in the replication cohort.

All maternal health characteristics were included in MANOVA analyses and subsequent multivariable linear/proportional odds regression models to reduce multiple testing and account for potential confounding. We evaluated the extent of correlation between maternal health characteristics in the discovery cohorts using Spearman’s rank correlation coefficient, and we did not find a high degree of correlation (>0.7 or <−0.7) (Figure S2).

In secondary analysis, we considered additional maternal health characteristics which may be important in shaping offspring health (e.g., prenatal stress, Social Vulnerability Index (SVI), residence, and type of insurance coverage). As these characteristics were only available for INSPIRE; we restricted this analysis to participants enrolled in this cohort. We evaluated associations between the above-mentioned parameters and newborn metabolite concentrations. For this secondary analysis, we utilized the same statistical plan carried out in the primary analysis. None of the maternal health characteristics were highly correlated (Figure S3).

To test the hypothesis that maternal health characteristics may increase the risk of later life metabolic dysfunction in offspring, we additionally explored relationships between metabolites significantly associated with maternal health characteristics in the primary and secondary analyses with child BMI from ages 1–3 years. This analysis was performed among subsets of the INSPIRE and Healthy Start cohorts with available weight and height measurements. For this analysis, we pooled INSPIRE and Healthy Start participants to increase power. We performed longitudinal linear mixed-effects regression modeling to assess associations between metabolite concentrations at birth and repeated child BMI measures from ages 1–3 years. In this analysis, we included participant ID as a random effect and adjusted for cohort, whether the recumbent length/standing height was estimated, age in days at BMI measurement, birth weight, gestational age, infant race, infant ethnicity, and sex. *p*-values for interactions between time (years) and metabolites were calculated using likelihood ratio tests. Data analyses were performed using R software, version 4.2.2 (R Foundation for Statistical Computing, Vienna, Austria). Additional details on methodology can be found in the Supplementary Material.

## 3. Results

**Figure 3:**
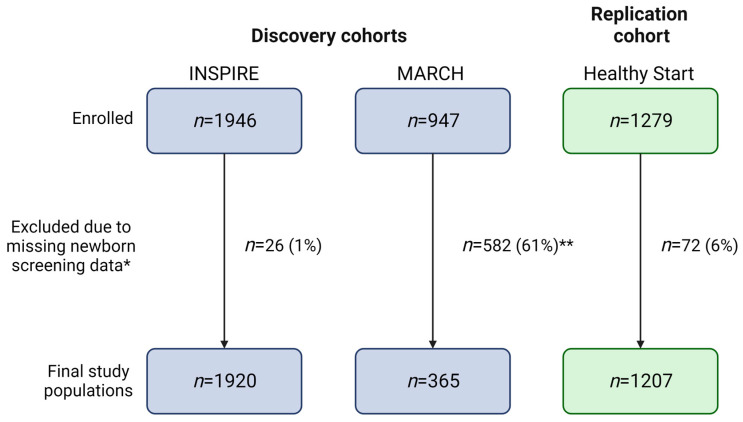
Flow diagram of study populations. Blue and green boxes are used to indicate number of participants in discovery and replication cohorts, respectively. * Newborn screening data may have been missing due to parental refusal of newborn screening, metabolite concentrations outside the normal range, or incomplete linkage. ** Obtaining participant consent for newborn screening metabolic data linkage is currently ongoing. This figure was created with BioRender.com.

Our final study populations included 1920 (INSPIRE, discovery cohort), 365 (MARCH, discovery cohort), and 1207 (Healthy Start, replication cohort) infants after linking NBS metabolic data to 99%, 39%, and 94% of infants in each cohort, respectively (Figure 3). As obtaining participant consent for NBS metabolic data linkage is currently ongoing for the MARCH cohort, we were only able to utilize data linked to 39% of the study population for the present study. We did not observe statistically significant differences between MARCH participants with NBS data and all MARCH participants (Table S5).

The prevalence of maternal health characteristics differed between the cohorts, with prenatal smoking, higher pre-pregnancy BMI, lower education, not being married, gestational diabetes, and delivery via c-section being more prevalent among INSPIRE and MARCH participants than among Healthy Start participants (Table 1). Maternal unemployment and higher maternal age at delivery were more prevalent among MARCH participants than INSPIRE and Healthy Start participants. Healthy Start participants had the lowest birth weights, and MARCH participants had the lowest gestational ages at birth (Table 2). Healthy Start was more ethnically diverse than INSPIRE and MARCH, with 29% of infants of Hispanic descent compared to 8% and 9%, respectively in the other two cohorts. The majority of INSPIRE and Healthy Start participants were White (76% and 70%), while MARCH was more racially diverse (53% White, 26% Black, 13% other, 8% missing). INSPIRE participants were enrolled after birth (mean age 2 months, standard deviation [SD] 2 months), while MARCH and Healthy Start participants were enrolled at birth.

**Figure 4:**
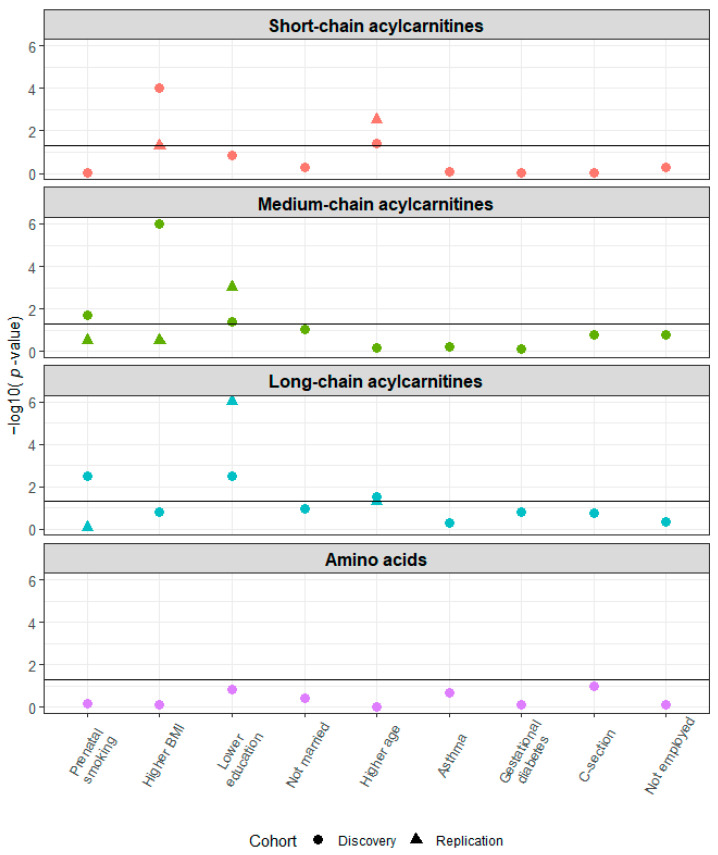
Higher age at delivery is associated with short-chain acylcarnitine concentrations at birth and lower education is associated with medium- and long-chain acylcarnitine concentrations at birth in the discovery (*n* = 2285) and replication (*n* = 1207) cohorts. Multivariate analysis of variance was used to assess the association between maternal health characteristics and established metabolite groups. This analysis was adjusted for birth weight, gestational age, infant race, infant ethnicity, sex, cohort (discovery phase only), and all other maternal health characteristics. The horizontal line indicates *p* = 0.05.

**Figure 5:**
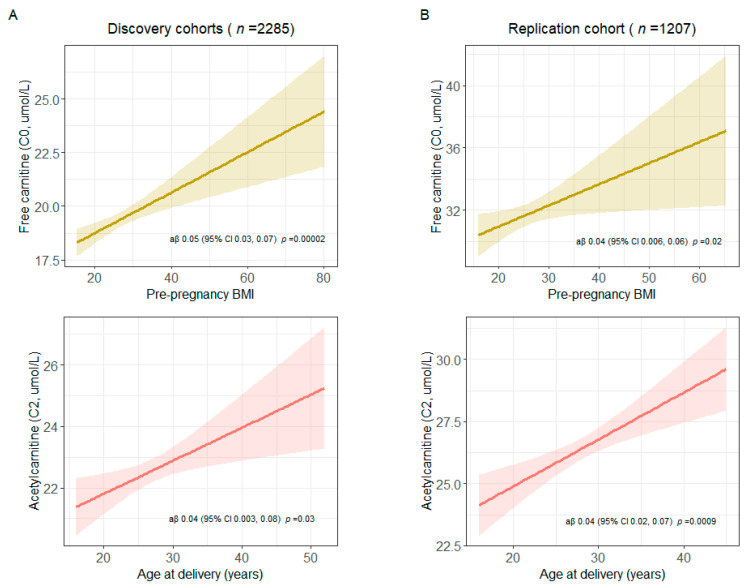
Higher pre-pregnancy BMI and higher age at delivery are associated with increased free carnitine (C0) and acetylcarnitine (C2) at birth, respectively, in both the **A**) discovery (*n* = 2285) and **B**) replication (*n* = 1207) cohorts. Multivariable linear regression was used to assess associations between higher BMI and C0 and higher age at delivery and C2. These analyses were adjusted for birth weight, gestational age, infant race, infant ethnicity, sex, cohort (discovery phase only), and all other maternal health characteristics. C0 and C2 were log-transformed. Point estimates were estimated for an 8.7 unit (interquartile range) increase in pre-pregnancy BMI and an 8-year increase in maternal age at delivery.

Several maternal health characteristics were associated with the *a priori* grouping of NBS metabolites as short-, medium-, and long-chain acylcarnitine concentrations at birth in the discovery cohorts (Figure 4). The associations of the following maternal health characteristics with NBS metabolite were also significant in the replication cohort: higher age at delivery and short-chain acylcarnitines; lower education and medium- and long-chain acylcarnitines. In analyses of the associations of maternal health characteristics and specific metabolites, we observed the following statistically significant associations in the discovery and replication cohorts: higher pre-pregnancy BMI and increased free carnitine (C0) concentration (discovery cohorts: aβ 0.05, 95% CI 0.03, 0.07; replication cohort: aβ 0.04, 95% CI 0.006, 0.06); higher age at delivery and increased acetylcarnitine (C2) concentration (discovery cohorts: aβ 0.04, 95% CI 0.003, 0.08; replication cohort: aβ 0.04, 95% CI 0.02, 0.07) (Figure 5 and Table S6). The results were unchanged after excluding women with potentially implausible pre-pregnancy BMIs > 50 (INSPIRE: *n* = 15 [1%], MARCH: *n* = 6 [2%], Healthy Start: *n* = 6 [0%]) (Figure S4).

In secondary analysis restricted to INSPIRE participants (n = 1920), we assessed associations between additional maternal health characteristics and newborn metabolite concentrations. Most participants reported no prenatal stress exposure (61%), resided in an urban environment (76%), and were on government insurance (54%) (Table S7). The mean SVI was 0.51 (SD 0.29). SVI was associated with long-chain acylcarnitine and amino acid concentrations; type of insurance coverage was associated with medium- and long-chain acylcarnitine concentrations; and residence was associated with medium-chain acylcarnitine concentrations (Figure S5). Prenatal stress exposure was not associated with any of the newborn metabolite groups. In analyses of the associations of prenatal stress exposure, SVI, type of insurance coverage, and residence and specific metabolites, we observed the following statistically significant associations: SVI and tetradecenoylcarnitine (C14:1), 3-hydroxypalmitoylcarnitine (C16-OH), and linoleoylcarnitine (C18:2); government insurance (vs. private insurance) and decanoylcarnitine (C10) and tetradecanoylcarnitine (C14); other insurance (vs. private insurance) and C0; and urban residence and C0, hexanoylcarnitine (C6), 3-hydroxytetradecanoylcarnitine (C14-OH), and C16-OH (Figure S6 and Table S8). Additionally, significant associations of pre-pregnancy BMI and C0 and maternal age at delivery and C2 remained after additional adjustment for prenatal stress exposure, SVI, type of insurance coverage, and residence.

**Figure 6:**
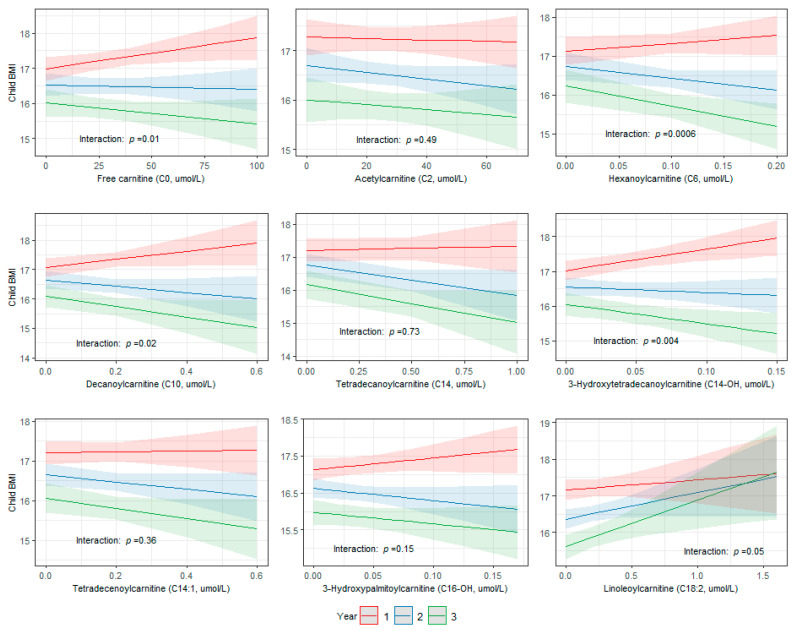
Associations between newborn metabolites that were associated with maternal health characteristics and child BMI were modified over time in a pooled subset of INSPIRE and Healthy Start children with available weight and height measurements (*n* = 2835). Longitudinal linear mixed-effects regression modeling was used to assess associations between metabolite concentrations at birth and repeated child BMI measures from ages 1–3 years, including participant ID as random effect and adjusting for cohort, whether the recumbent length/standing height was estimated, age in days at BMI measurement, birth weight, gestational age, infant race, infant ethnicity, and sex. *p*-values for interactions between time (years) and metabolites were calculated using likelihood ratio tests.

We then assessed the association between newborn metabolites that were significantly associated with maternal health characteristics in the primary and secondary analyses (C0, C2, C6, C10, C14, C14-OH, C14:1, C16-OH, and C18:2) and child BMI from ages 1–3 years in a pooled subset of INSPIRE and Healthy Start children with available weight and height measurements. Of the 2835 children in the pooled subset, 2122 (75%; *n* = 1130 INSPIRE, *n* = 992 Healthy Start), 2176 (77%; *n* = 1161 INSPIRE, *n* = 1015 Healthy Start), and 1272 (45%; *n* = 398 INSPIRE, *n* = 874 Healthy Start) children had weight and height measurements at ages 1, 2, and 3 years, respectively. The mean BMI was 17 (SD 2) at 1 year, 17 (SD 2) at 2 years, and 16 (2) at 3 years (Figure S7). The associations between C0, C6, C10, and C14-OH and child BMI were modified over time (interaction: *p* < 0.05) (Figure 6). The strongest effects were present at year 1, and the associations waned over time.

## 4. Discussion

In this multi-cohort study, we identified maternal health characteristics associated with newborn metabolites. We identified and replicated relationships between higher pre-pregnancy BMI and increased C0 concentration at birth and higher age at delivery and increased C2 concentration at birth in discovery and replication cohorts. We also observed relationships between SVI, type of insurance coverage, and residence and C0 and medium- and long-chain acylcarnitine (C6, C10, C14, C14-OH, C14:1, C16-OH, and C18:2) concentrations at birth within a discovery cohort. Additionally, we showed that associations between metabolites associated with maternal health characteristics (C0, C6, C10, and C14-OH) and child BMI were modified from ages 1–3 years. These findings suggest that maternal health characteristics may impact fetal metabolic programming, as measured by NBS metabolites, potentially influencing later life child growth patterns. Future studies should perform formal mediation analyses to further explore the potential biologic pathways through which maternal health characteristics may impact child BMI.

Few studies have assessed associations between maternal complications of pregnancy and environmental stressors and newborn metabolite concentrations. McCarthy et al. showed significant associations between socioeconomic status—comprised of Medicaid coverage; Women, Infants, and Children (WIC) supplemental nutrition receipt; and having a high school education or less—and 41 of 42 targeted NBS (blood spot) metabolites in a California birth cohort [21]. Lowe et al. found associations between increased second-trimester BMI and insulin resistance and increased cord blood concentrations of branched-chain amino acids and their metabolic byproducts [22]. In a cohort of preterm infants, Ryckman et al. found associations between preeclampsia and increased concentrations of certain metabolites quantified from NBS panels (alanine, C0, C2, octenotylcarnitine [C8:1], and C18:2) [23]. We have extended these findings by comprehensively demonstrating and replicating associations between maternal health characteristics and newborn metabolite concentrations and the association of these metabolites with childhood growth patterns. While previous studies have shown associations between newborn metabolites and childhood growth [24], overweight [24], and obesity [25], this study provides additional insights on the maternal factors and potential in utero pathways underlying the relationship observed between newborn metabolism and subsequent child metabolic dysfunction.

We identified associations between several maternal health characteristics (pre-pregnancy BMI, age at delivery, SVI, type of insurance coverage, and residence) and concentrations of free carnitine (C0) and acylcarnitines (C2, C6, C10, C14, C14-OH, C14:1, C16-OH, and C18:2) at birth. C0 and its acylated derivatives (i.e., acylcarnitines), known as the carnitine pool, play a vital role in mitochondrial function and energy production [9]. Humans oxidize extensive amounts of fats to guarantee continuous energy supply [26]. C0 is essential for fatty acid oxidation as it is the primary shuttle for long-chain acylcarnitines from the cytosol into the mitochondrion [26]. Acylcarnitines are formed from carnitine and acyl-CoAs in the mitochondria and are the transport form of fatty acids in the plasma [27,28]. External stressors have been shown to impact the carnitine pool. Decreased C0 levels are found in individuals with obesity and insulin resistance due to compromised mitochondrial function and fatty acid oxidation [29]. The association that we observed in the present study between higher pre-pregnancy BMI and increased C0 concentration at birth may be due to a rebound effect, with increased C0 in the newborn due to diminished supply in utero. Increases in C2 are associated with stress/trauma [30]. Increased maternal age at delivery is also associated with increased prenatal stress [31,32], anxiety [31], and complications of delivery [33], suggesting one possible explanation for the observed increased C2 in offspring of older women. Socioeconomic status and stress can impact food access [34] and eating behaviors [35,36], which may lead to metabolite fluctuations [37]. We observed relationships between SVI and type of insurance and concentrations of several metabolites at birth, which may additionally reflect food access, eating behaviors, and metabolism. Air pollutants contribute to mitochondrial dysfunction [38], which may account for the relationships observed between urban residence and free carnitine and several acylcarnitines.

As the carnitine pool is tightly regulated, changes in plasma carnitine and acylcarnitine concentrations may contribute to metabolic dysfunction and disease [9]. This is supported by our findings of associations between metabolite concentrations at birth and subsequent child growth patterns. We observed that some acylcarnitine concentrations at birth (C0, C6, C10, and C14-OH) were associated with maternal health characteristics and child BMI at ages 1–3 years. However, other acylcarnitines associated with maternal health characteristics (C2, C14, C14:1, C16-OH, and C18:2) were not associated with child BMI at ages 1–3 years. C2, C14, C14:1, C16-OH, and C18:2 may be associated with other important metabolic parameters in childhood, such as central adiposity, waist circumference, and triglyceride and fasting insulin levels, and these relationships should be assessed in future studies.

Our study has many important strengths, including our large sample sizes from diverse, longitudinal birth cohorts with rich phenotypic data and our replication of findings in an independent birth cohort. In addition, harmonization of NBS data across cohorts was possible because processing of NBS samples is regulated by the CDC’s Newborn Screening Quality Assurance Program and consensus guidelines on the use of MS/MS analysis in NBS developed by the Clinical and Laboratory Standards Institute ensure standardization across states [14,39]. Lastly, we employed a rigorous *a priori* two-stage analytic plan to reduce multiple testing of a large NBS metabolite panel.

There are also some limitations of our study. We could only assess maternal health characteristics that were ascertained and harmonizable across cohorts. We assessed the association between additional maternal health characteristics (prenatal stress, SVI, residence, and type of insurance coverage) and newborn metabolite concentrations within the INSPIRE cohort but were unable to replicate these results due to lack of currently available information on these characteristics within MARCH and Healthy Start cohorts. While we were able to harmonize maternal health characteristics across the cohorts in this study, measurement error is possible due to the use of different means and timing of ascertainment of the maternal health characteristics across cohorts. We utilized maternal race and ethnicity as surrogate measures for infant race and ethnicity for participants in whom these data were unavailable. Although this is an imperfect measure, the concordances between maternal race and ethnicity and infant race and ethnicity were high, and use of this surrogate measure is unlikely to have impacted the results. Although NBS data were only available for 39% of MARCH participants enrolled in this birth cohort as obtaining participant consent for NBS metabolic data linkage is currently ongoing, we did not observe differences between MARCH participants with NBS data and all MARCH participants. This suggests that selection bias would not have impacted our results. We chose to only include child BMI measures from ages 1–3 years in the present study. Future studies should assess the association between newborn metabolites associated with maternal health characteristics and child BMI beyond age 3 years to further explore relationships with child growth patterns.

## 5. Conclusions

In this multi-cohort study, we identified and replicated associations between maternal health characteristics and newborn metabolite concentrations. We further demonstrated that metabolites associated with maternal health characteristics were also associated with child growth patterns. These findings may provide important insights on potential biologic pathways through which maternal health characteristics may impact fetal metabolic programming and later childhood metabolic dysfunction and growth patterns.

## Figures and Tables

**Table 1:** Maternal health characteristics of the study populations with linked newborn screening metabolic data prior to multiple imputation.

	Cohort			
Maternal Health Characteristic	INSPIRE	MARCH	Healthy Start	*p*-Value ^a^
Sample size	1920	365	1207	
Prenatal smoking, *n* (%)	345 (18)	41 (11)	90 (7)	<0.001 *
Missing, *n* (%)	2 (0)	47 (13)	0 (0)	
Pre-pregnancy BMI ^b^, mean (SD)	27 (7)	29 (8)	26 (6)	<0.001 *
Missing, *n* (%)	72 (4)	43 (12)	0 (0)	
Education, *n* (%)				<0.001 *
<High school	153 (8)	31 (8)	166 (14)	
High school degree	524 (27)	68 (19)	215 (18)	
Some college ^b^	572 (30)	121 (33)	271 (22)	
≥College degree ^b^	670 (35)	100 (27)	555 (46)	
Missing, *n* (%)	1 (0)	45 (12)	0 (0)	
Occupational status, *n* (%)				0.001 *
Not employed	669 (35)	81 (22)	388 (32)	
Employed	1251 (65)	239 (65)	682 (57)	
Missing, *n* (%)	0 (0)	45 (12)	137 (11)	
Marital status, *n* (%)				<0.001 *
Not married	816 (43)	177 (48)	447 (37)	
Married	1104 (58)	142 (39)	755 (63)	
Missing, *n* (%)	0 (0)	46 (13)	5 (0)	
Age at delivery (years) ^b^, mean (SD)	27 (5)	29 (6)	28 (6)	<0.001 *
Missing, *n* (%)	0 (0)	71 (19)	10 (1)	
Asthma, *n* (%)	372 (19)	67 (18)	197 (16)	0.05
Missing, *n* (%)	1 (0)	46 (13)	1 (0)	
Gestational diabetes, *n* (%)	126 (7)	23 (6)	47 (4)	0.01 *
Missing, *n* (%)	0 (0)	71 (19)	96 (8)	
C-section, *n* (%)	600 (31)	105 (29)	250 (21)	<0.001 *
Missing, *n* (%)	0 (0)	71 (19)	27 (2)	

BMI—body mass index; SD—standard deviation. * *p* < 0.05, ^a^ *p*-value for comparisons between cohorts calculated using Kruskal–Wallis or Pearson χ^2^ test, as appropriate. ^b^ Some college was defined as no degree, Associate’s degree, or trade/technical/vocational training; ≥college degree was defined as Bachelor’s, Master’s, Professional, or Doctorate degree.

**Table 2:** Infant characteristics of the study populations with linked newborn screening metabolic data prior to multiple imputation.

	Cohort			
Infant Characteristic	INSPIRE	MARCH	Healthy Start	*p*-Value ^a^
Sample Size	1920	365	1207	
Birth weight (grams) ^b^, mean (SD)	3432 (461)	3225 (577)	3218 (526)	<0.001 *
Missing, *n* (%)	0 (0)	71 (19)	14 (1)	
Gestational age (weeks) ^b^, mean (SD)	39 (1)	38 (2)	39 (2)	<0.001 *
Missing, *n* (%)	0 (0)	71 (19)	9 (1)	
Race, *n* (%)				<0.001 *
White	1451 (76)	195 (53)	850 (70)	
Black	353 (18)	94 (26)	156 (13)	
Other	116 (6)	46 (13)	201 (17)	
Missing, *n* (%)	0 (0)	30 (8)	0 (0)	
Hispanic ethnicity, *n* (%)	161 (8)	34 (9)	356 (29)	<0.001 *
Missing, *n* (%)	4 (0)	30 (8)	0 (0)	
Male sex, *n* (%)	1009 (53)	137 (38)	606 (50)	0.16
Missing, *n* (%)	0 (0)	71 (19)	24 (2)	
Age at enrollment (months) ^b^, mean (SD)	2 (2)	0 (0) ^b^	0 (0) ^b^	<0.001 *
Missing, *n* (%)	0 (0)	0 (0)	0 (0)	

SD—standard deviation. * *p* < 0.05, ^a^ *p*-values for comparisons between cohorts calculated using Kruskal–Wallis or Pearson χ^2^ test, as appropriate. ^b^ Infants were enrolled at birth.

## Data Availability

The data presented in this study are available on request from the corresponding author. The data are not publicly available due to linkage to potentially identifying and sensitive patient information of participants in three National Institutes of Health (NIH) Environmental Influences on Child Health Outcomes (ECHO) Program birth cohorts. The dataset includes confidential newborn screening data that cannot be shared without individual approvals from the Tennessee Department of Health, Michigan Department of Health and Human Services, and Colorado Department of Public Health and Environment Institutional Review Boards. Access to confidential ECHO data requires written authorization from the ECHO study sponsor, the ECHO Program, and a data request submitted to the ECHO PI.

## Supplementary Materials

The following supporting information can be downloaded at: https://www.mdpi.com/article/10.3390/metabo13040510/s1, Supplementary Material (includes additional methodological details and supplementary figure legends); Table S1: Newborn screening (NBS) metabolites measured in each cohort; Table S2: Availability of maternal health characteristics by cohort; Table S3: Newborn screening metabolite concentrations (*n* = 31) by cohort; Table S4: Pre-specified metabolite groups included in stage one of the statistical plan; Table S5: Comparison of characteristics for MARCH participants with newborn screening (NBS) metabolic data and all MARCH participants; Table S6: Associations between maternal health characteristics and metabolite concentrations at birth in the discovery (*n* = 2285) and replication (*n* = 1207) cohorts; Table S7: Additional maternal health characteristics of INSPIRE participants with linked newborn screening metabolic data (*n* = 1920); Table S8: Associations between prenatal stress exposure, Social Vulnerability Index (SVI), type of insurance coverage, and residence and metabolite concentrations at birth in INSPIRE (*n* = 1920); Figure S1: Newborn screening metabolite distributions in the discovery cohorts (INSPIRE: *n* = 1920, MARCH: *n* = 365); Figure S2: Correlation between maternal health characteristics in the discovery cohorts (*n* = 2285); Figure S3: Correlation between prenatal stress exposure, Social Vulnerability Index, residence, and type of insurance and all other maternal health characteristics in INSPIRE (*n* = 1920); Figure S4: Higher pre-pregnancy BMI and higher age at delivery are associated with increased free carnitine (C0) and acetylcarnitine (C2) at birth, respectively, in both the discovery (*n* = 2264) and replication (*n* = 1201) cohorts after excluding women with potentially implausible pre-pregnancy BMIs (>50); Figure S5: Social Vulnerability Index (SVI) is associated with long-chain acylcarnitine and amino acid concentrations at birth, type of insurance coverage is associated with medium- and long-chain acylcarnitine concentrations at birth, and residence is associated with medium-chain acylcarnitine concentrations at birth in INSPIRE (*n* = 1920); Figure S6: Statistically significant associations between Social Vulnerability Index, type of insurance coverage, and residence and newborn metabolite concentrations in INSPIRE (*n* = 1920); Figure S7: Distribution of BMI measurements at ages 1, 2, and 3 years in INSPIRE (*n* = 1692) and Healthy Start (*n* = 1143).
