# Supplementary material for: The Associations of Maternal Health Characteristics, Newborn Metabolite Concentrations, and Child Body Mass Index among US Children in the ECHO Program
Source: Metabolites. 2023 Apr 1;13(4):510. doi: 10.3390/metabo13040510 (PMC10144800; doi:10.3390/metabo13040510)
Supplement: Supplementary file 1 [file metabolites-13-00510-s001.zip › Supplementary_Material- Clean.pdf]

The associations of maternal health characteristics, newborn metabolite concentrations, and child body mass index among US children in the ECHO Program

Brittney M. Snyder, Tebeb Gebretsadik, Nina B. Rohrig, Pingsheng Wu, William D. Dupont, Dana M. Dabelea, Rebecca C. Fry, Susan V. Lynch, Cindy T. McEvoy, Nigel S. Paneth, Kelli K. Ryckman, James E. Gern, Tina V. Hartert and on behalf of program collaborators for Environmental influences on Child Health Outcomes

Supplementary Material

Methods 2

Study design and populations 2

Newborn screening metabolic data collection 2

Maternal health characteristics and covariate ascertainment 4

Child BMI ascertainment 5

Methods Table S1 6

Methods Table S2 7

Statistical analysis 9

References 13

Supplementary figure legends 16

Methods

Study design and populations

This multi-site study included three birth cohorts from the National Institutes of Health (NIH) Environmental influences on Child Health Outcomes (ECHO) Program (https://echochildren.org/). The INfant Susceptibility to Pulmonary Infections and asthma following RSV Exposure (INSPIRE) cohort included term, healthy infants enrolled shortly after birth (2012-2014) from pediatric practices located in middle Tennessee. The Michigan Archive for Research on Child Health (MARCH) cohort included infants enrolled in pregnancy in a population-based sample from 21 prenatal clinics and delivering in 11 birth hospitals in the lower peninsula of Michigan from 2017-2022. The Healthy Start cohort included infants born to women at the University of Colorado Hospital from 2010-2014. We linked NBS blood metabolic data with each of these cohorts and included enrolled infants with linked NBS blood metabolic data.

Newborn screening metabolic data collection

Newborn screening (NBS) is a public health initiative aimed at screening every infant at birth for inborn errors of metabolism with the goal of initiating treatment prior to symptom onset [1]. NBS metabolic data include targeted measurement of free carnitine, acylcarnitines, and amino acids. Collection of blood spot cards for NBS is standardized, requiring collection by a health care professional within 24-48 hours after birth and sent to the respective state laboratory for routine testing [1]. Tandem mass spectrometry (MS/MS) was then used to quantitively measure metabolite concentrations using the calculated ratio of the signal from each metabolite to the signal from the known amount of internal standard [2]. Quantified results were stored on

state public health department servers. Most states screen for the 35 conditions recommended by the US Health Resources & Services Administration. However, the conditions, and corresponding metabolites, included on each state's NBS panel can vary based on the state public health department's assessment of the net benefit of screening, availability of effective treatments, and screening capabilities of the state [3].

Existing NBS metabolic data were provided for infants enrolled in the cohorts by the NBS programs at the Tennessee Department of Health, Michigan Department of Health and Human Services, and Colorado Department of Public Health and Environment. Metabolites measured in each cohort are listed in **Table S1**. Data were provided for infants who did not screen positive for any inherited disorder (i.e., metabolite concentrations were within the normal range, representing >99% of infants in the US [4]) to reduce the risk of potential participant identification and remove skewed metabolic profiles due to inborn errors of metabolism. We then linked the metabolic data with demographic and clinical data from each of the cohorts. Repeat blood spot testing results were provided for a small subset of infants based on state protocols (INSPIRE: n=37 [2% of enrolled participants], MARCH: n= 0, Healthy Start: n=0). For infants with two viable samples collected (INSPIRE: n=30), the average value of each metabolite was calculated and used in the analyses. For the remaining seven INSPIRE infants in whom the primary collection occurred outside of the protocol time frame (i.e., <24 hours after birth) or the sample was processed >10 days after collection, the value for the second specimen was used [5] .

Maternal health characteristics and covariate ascertainment

We assessed several maternal health characteristics based on cohort availability. Maternal health characteristics (e.g., prenatal smoking [yes, no], pre-pregnancy body mass index [BMI, continuous], education [<high school, high school degree, some college, ≥college degree], occupational status [not employed, employed], marital status [not married, married], age at delivery [years, continuous], asthma [yes, no], gestational diabetes [yes, no], and mode of delivery [c-section, vaginal]) were ascertained from questionnaires administered at enrollment for INSPIRE participants, birth certificates and questionnaires administered during pregnancy for MARCH participants, and medical records at delivery and questionnaires administered during pregnancy for Healthy Start participants (**Table S2**).

Covariates (e.g., birth weight [grams, continuous], gestational age [weeks, continuous], infant race [White, Black, other], infant ethnicity [Hispanic, not Hispanic], and sex [male, female]) were collected from enrollment questionnaires for INSPIRE participants. Birth weight, gestational age, and sex were ascertained from birth certificates for MARCH participants, while infant race and ethnicity were collected from questionnaires administered at infant age 3-months. We utilized maternal race and ethnicity as surrogate measures of infant race and ethnicity for the 33% and 32% of MARCH participants who were missing infant race and ethnicity, respectively (88% concordance for race and 94% concordance ethnicity among those with both maternal and infant race and ethnicity data available). For Healthy Start participants, sex was ascertained from delivery questionnaires. Infant race and ethnicity were ascertained from questionnaires administered at infant age 6-months. Maternal race and ethnicity were used as proxies for 34% and 32% of infants with missing race and ethnicity, respectively. As maternal race and maternal ethnicity were not provided as separate variables (i.e., infant race and ethnicity were filled in

with maternal race and ethnicity if missing), we could not calculate concordance between maternal and infant race and ethnicity for Healthy Start participants. Birth weight was derived from several sources using the following hierarchy: 1) newborn medical record abstraction, 2) newborn physical exam performed within a week after birth, 3) self-reported at delivery interview, 4) self-reported at infant 6-month visit, and 5) self-reported at infant 18-month visit. Gestational age was ascertained from medical records for 96% of participants and delivery questionnaires for 3% of participants (1% of participants were missing information on gestational age).

Child BMI ascertainment

We ascertained child BMI at ages 1, 2, and 3 years in subsets of the INSPIRE and Healthy Start cohorts with available weight and height measurements. Child BMI was ascertained through medical record abstraction for participants in the Healthy Start cohort and from medical records and/or study visits for participants in the INSPIRE cohort. If more than one weight and height measurement was collected during the year, we used the latest measurement. All weight and height measurements were collected on the same date, within each year, for Healthy Start children. We calculated estimated recumbent length/standing height at weight measurement date for INSPIRE children with lengths/heights and weights measured on different days within each year (year 1: n=60, year 2: n=186, year 3: n=17) using World Health Organization (WHO) growth charts[6] for children age <2 years (recumbent length) and Centers for Disease Control (CDC) growth charts[7] for children age 2-3 years (standing height) [8].

For children age <2 years, we first calculated recumbent length z-scores using the following equation [9]:

$$z - score = \frac{(Length/M)^L - 1}{L * S}$$

where M is the median, L is power, and S is variation (**Methods Table S1**). For this calculation, we used age at length measurement.

Methods Table S1. Length-for-age percentiles, 0-23 months, WHO growth standards.

Girls				Boys			
**Age [months]**	**Power [L]**	**Median [M]**	**Variation [S]**	**Age [months]**	**Power [L]**	**Median [M]**	**Variation [S]**
0	1	49.1477	0.03790	0	1	49.8842	0.03795
1	1	53.6872	0.03640	1	1	54.7244	0.03557
2	1	57.0673	0.03568	2	1	58.4249	0.03424
3	1	59.8029	0.03520	3	1	61.4292	0.03328
4	1	62.0899	0.03486	4	1	63.8860	0.03257
5	1	64.0301	0.03463	5	1	65.9026	0.03204
6	1	65.7311	0.03448	6	1	67.6236	0.03165
7	1	67.2873	0.03441	7	1	69.1645	0.03139
8	1	68.7498	0.03440	8	1	70.5994	0.03124
9	1	70.1435	0.03444	9	1	71.9687	0.03117
10	1	71.4818	0.03452	10	1	73.2812	0.03118
11	1	72.7710	0.03464	11	1	74.5388	0.03125
12	1	74.0150	0.03479	12	1	75.7488	0.03137
13	1	75.2176	0.03496	13	1	76.9186	0.03154
14	1	76.3817	0.03514	14	1	78.0497	0.03174
15	1	77.5099	0.03534	15	1	79.1458	0.03197
16	1	78.6055	0.03555	16	1	80.2113	0.03222
17	1	79.6710	0.03576	17	1	81.2487	0.03250
18	1	80.7079	0.03598	18	1	82.2587	0.03279
19	1	81.7182	0.03620	19	1	83.2418	0.03310
20	1	82.7036	0.03643	20	1	84.1996	0.03342
21	1	83.6654	0.03666	21	1	85.1348	0.03376
22	1	84.6040	0.03688	22	1	86.0477	0.03410
23	1	85.5202	0.03711	23	1	86.9410	0.03445

We then used z-scores to back-calculate length at age of weight measurement using the following equation:

$$Length = M(1 + ((L * S) * z - score))$$

Methods Table S1 was used again in this calculation. However, age at weight measurement was used instead of age at length measurement (as was done for the prior calculation). This estimated recumbent length at weight measurement date was then used in the analyses.

For children age 2-3 years, we calculated the standing height z-scores using the following equation [10,11]:

$$z - score = \frac{(Length/M)^L - 1}{L * S}$$

where M is the median, L is power, and S is variation (**Methods Table S2**). For this calculation, we used age at height measurement.

Methods Table S2. Stature-for-age percentiles, 2-20 years, CDC growth standards.

Girls				Boys			
**Age [months]**	**Power [L]**	**Median [M]**	**Variation [S]**	**Age [months]**	**Power [L]**	**Median [M]**	**Variation [S]**
24	1.051272912	85.3973169	0.040859727	24	1.00720807	86.86160934	0.040395626
25	1.041951175	86.29026318	0.041142161	25	0.837251351	87.65247282	0.040577525
26	1.012592236	87.15714182	0.041349399	26	0.681492975	88.42326434	0.040723122
27	0.970541909	87.9960184	0.041500428	27	0.538779654	89.17549228	0.040833194
28	0.921129988	88.8055115	0.041610508	28	0.407697153	89.91040853	0.040909059
29	0.868221392	89.58476689	0.041691761	29	0.286762453	90.62907762	0.040952433
30	0.81454413	90.33341722	0.04175368	30	0.174489485	91.33242379	0.04096533
31	0.761957977	91.0515436	0.041803562	31	0.069444521	92.02127167	0.040949976
32	0.711660228	91.7396352	0.041846882	32	-0.029720564	92.69637946	0.040908737
33	0.664323379	92.39854429	0.041887626	33	-0.124251789	93.35846546	0.040844062
34	0.620285102	93.02945392	0.041928568	34	-0.215288396	94.00822923	0.040758431
35	0.57955631	93.63382278	0.041971514	35	-0.30385434	94.64636981	0.040654312
36	0.54198094	94.21335709	0.042017509	36	-0.390918369	95.27359106	0.04053412
37	0.511429832	94.79643239	0.042104522	37	-0.254801167	95.91474929	0.040572876
38	0.482799937	95.37391918	0.042199507	38	-0.125654535	96.54734328	0.04061691
39	0.455521041	95.94692677	0.042300333	39	-0.00316735	97.17191309	0.040666414
40	0.429150288	96.51644912	0.042405225	40	0.11291221	97.78897727	0.040721467
41	0.403351725	97.08337211	0.042512706	41	0.222754969	98.3990283	0.040782045
42	0.377878239	97.6484807	0.042621565	42	0.326530126	99.00254338	0.040848042
43	0.352555862	98.21246579	0.042730809	43	0.42436156	99.599977	0.040919281
44	0.327270297	98.77593069	0.042839638	44	0.516353108	100.191764	0.040995524
45	0.301955463	99.33939735	0.042947412	45	0.602595306	100.7783198	0.041076485
46	0.276583851	99.9033122	0.043053626	46	0.683170764	101.3600411	0.041161838
47	0.251158446	100.4680516	0.043157889	47	0.758158406	101.9373058	0.041251224
48	0.225705996	101.033927	0.043259907	48	0.827636736	102.5104735	0.041344257
49	0.20027145	101.6011898	0.043359463	49	0.891686306	103.0798852	0.041440534

50	0.174913356	102.1700358	0.043456406	50	0.95039153	103.645864	0.041539635
51	0.149700081	102.7406094	0.043550638	51	1.003830006	104.208713	0.041641136
52	0.12470671	103.3130077	0.043642107	52	1.05213569	104.7687256	0.041744602
53	0.100012514	103.8872839	0.043730791	53	1.0953669	105.3261638	0.041849607
54	0.075698881	104.4634511	0.043816701	54	1.133652119	105.8812823	0.041955723
55	0.051847635	105.0414853	0.043899867	55	1.167104213	106.4343146	0.042062532
56	0.02853967	105.6213287	0.043980337	56	1.195845353	106.9854769	0.042169628
57	0.005853853	106.2028921	0.044058171	57	1.220004233	107.534968	0.042276619
58	-0.016133871	106.7860583	0.04413344	58	1.239715856	108.0829695	0.042383129
59	-0.037351181	107.3706841	0.044206218	59	1.255121285	108.6296457	0.042488804
60	-0.057729947	107.9566031	0.044276588	60	1.266367398	109.1751441	0.042593311
61	-0.077206672	108.5436278	0.044344632	61	1.273606657	109.7195954	0.042696342
62	-0.09572283	109.1315521	0.044410436	62	1.276996893	110.2631136	0.042797615
63	-0.113225128	109.7201531	0.044474084	63	1.276701119	110.8057967	0.042896877
64	-0.129665689	110.3091934	0.044535662	64	1.272887366	111.3477265	0.042993904
65	-0.145002179	110.8984228	0.044595254	65	1.265728536	111.8889694	0.043088503
66	-0.159197885	111.4875806	0.044652942	66	1.255402281	112.4295761	0.043180513
67	-0.172221748	112.0763967	0.044708809	67	1.242090871	112.9695827	0.043269806
68	-0.184048358	112.6645943	0.044762936	68	1.225981067	113.5090108	0.043356287
69	-0.194660215	113.2518902	0.044815402	69	1.207263978	114.0478678	0.043439893
70	-0.204030559	113.8380006	0.044866288	70	1.186140222	114.5861486	0.043520597
71	-0.212174408	114.4226317	0.044915672	71	1.162796198	115.1238315	0.043598407
72	-0.219069129	115.0054978	0.044963636	72	1.137442868	115.6608862	0.043673359
73	-0.224722166	115.5863089	0.045010259	73	1.110286487	116.1972691	0.043745523
74	-0.229140412	116.1647782	0.045055624	74	1.081536236	116.732925	0.043815003
75	-0.232335686	116.7406221	0.045099817	75	1.05140374	117.2677879	0.043881929
76	-0.234324563	117.3135622	0.045142924	76	1.020102497	117.8017819	0.043946461
77	-0.235128195	117.8833259	0.045185036	77	0.987847213	118.3348215	0.044008785
78	-0.234772114	118.4496481	0.045226249	78	0.954853043	118.8668123	0.044069112
79	-0.233286033	119.0122722	0.045266662	79	0.921334742	119.397652	0.044127675
80	-0.230703633	119.5709513	0.045306383	80	0.887505723	119.9272309	0.044184725
81	-0.227062344	120.1254495	0.045345524	81	0.85357703	120.455433	0.044240532
82	-0.222403111	120.6755427	0.045384203	82	0.819756239	120.9821362	0.044295379
83	-0.216770161	121.22102	0.045422551	83	0.786246296	121.5072136	0.044349559
84	-0.210210748	121.7616844	0.045460702	84	0.753244292	122.0305342	0.044403374
85	-0.202774891	122.2973542	0.045498803	85	0.720940222	122.5519634	0.04445713
86	-0.194515104	122.827864	0.045537012	86	0.689515708	123.0713645	0.044511135
87	-0.185486099	123.3530652	0.045575495	87	0.659142731	123.588599	0.044565693
88	-0.175744476	123.8728276	0.045614432	88	0.629997853	124.1035312	0.044621104
89	-0.165348396	124.38704	0.045654016	89	0.602203984	124.6160161	0.044677662
90	-0.15435722	124.8956114	0.04569445	90	0.575908038	125.1259182	0.044735646
91	-0.142831123	125.398472	0.045735953	91	0.55123134	125.6331012	0.044795322
92	-0.130830669	125.895574	0.045778759	92	0.528279901	126.1374319	0.044856941
93	-0.118416354	126.3868929	0.045823114	93	0.507143576	126.6387804	0.04492073
94	-0.105648092	126.8724284	0.04586928	94	0.487895344	127.1370217	0.044986899
95	-0.092584657	127.3522056	0.045917535	95	0.470590753	127.6320362	0.045055632
96	-0.079283065	127.8262759	0.045968169	96	0.455267507	128.1237104	0.045127088
97	-0.065797888	128.2947187	0.04602149	97	0.441945241	128.6119383	0.045201399
98	-0.0521805	128.757642	0.046077818	98	0.430625458	129.096622	0.045278671
99	-0.03847825	129.2151839	0.046137487	99	0.421291648	129.5776723	0.045358979
100	-0.024733545	129.6675143	0.046200842	100	0.413909588	130.0550101	0.045442372

We then used z-scores to back-calculate height at age of weight measurement using the following equation:

$$Height = M(((z - score(L * S)) + 1)^{1/L})$$

Methods Table S2 was used again in this calculation. However, age at weight measurement was used instead of age at height measurement (as was done for the prior calculation). This estimated standing height at weight measurement date was then used in the analyses.

Statistical analysis

We compared maternal characteristics, infant characteristics, and metabolite concentrations between the cohorts using Kruskal-Wallis or Pearson χ^2 test, as appropriate. We used multiple imputation (n=5 iterations) using Fully Conditional Specification (FCS) implemented by the Multivariate Imputation by Chained Equations (MICE) algorithm for each cohort separately to estimate possible values for missing data [12]. Each variable had its own imputation model (continuous variables: predictive mean matching; binary variables: logistic regression; unordered categorical variables: polytomous logistic regression; ordered categorical variables: proportional odds). Outcome was included in the imputation, but analyses were restricted to participants in whom we had observed outcome data [13]. All analyses were performed using multiply imputed datasets. For the primary analysis, we pooled the cohorts with the largest and smallest number of infants with linked NBS metabolic data in a discovery phase (INSPIRE and MARCH), and we utilized the Healthy Start cohort in a replication phase to have similar regression power. Metabolites measured in both the INSPIRE and MARCH cohorts were included in the analysis (n=31, **Table S1**). NBS metabolite concentrations for the study populations are shown in **Table S3**.

Our *a priori* statistical plan consisted of a two-stage process (**Figure 2).** In stage one, we assessed the associations between maternal health characteristics and established metabolite

groups[14] (short-, medium-, and long-chain acylcarnitines and amino acids [**Table S4**]) in the discovery cohorts using multivariate analysis of variance (MANOVA), adjusting for cohort, birth weight, gestational age, infant race, infant ethnicity, and sex. Assessing global associations between maternal health characteristics and metabolite groups through MANOVA, compared to each metabolite separately, helped reduce the multiple testing burden. As MANOVA assumes interval measurement of dependent variables, only metabolites with continuous distributions (n=26) were included in these pre-specified groups (**Figure S1)**. Metabolites were log-transformed to meet regression assumptions. As this was an exploratory analysis, p-values <0.05 were considered statistically significant. We then repeated this analysis in the replication cohort, adjusting for the same covariates (excluding cohort).

In stage two, for maternal health characteristic-metabolite group associations that were statistically significant in both the discovery and replication cohorts, we assessed the relationships between maternal health characteristics and each metabolite, including free carnitine (C0) which did not fit into one of the pre-specified metabolite groups, using multivariable linear regression We also assessed the relationship between maternal health characteristics and n=5 metabolites with ordinal distributions (tiglylcarnitine [C5:1], decadienoylcarnitine [C10:2], 3-hydroxytetradecanoylcarnitine [C14-OH], 3-hydroxypalmitoylcarnitine [C16-OH], and 3-hydroxyoleoylcarnitine [C18:1-OH] [**Figure S1]**), which did not meet MANOVA assumptions, using proportional odds regression. For maternal health characteristic-metabolite associations that remained statistically significant in discovery cohorts, we repeated this analysis in the replication cohort.

All maternal health characteristics were included in MANOVA analyses and subsequent multivariable linear/proportional odds regression models to reduce multiple testing and account

for potential confounding. We calculated point estimates and confidence intervals for interquartile range (IQR) difference increases in age at delivery and pre-pregnancy BMI and a one-unit decrease in education. We evaluated the extent of correlation between maternal health characteristics in the discovery cohorts using Spearman's rank correlation coefficient, and we did not find a high degree of correlation (>0.7 or <-0.7) (**Figure S2**).

In secondary analysis, we considered additional maternal health characteristics which may be important in shaping offspring health (e.g., prenatal stress, Social Vulnerability Index (SVI), residence, and type of insurance coverage). As these characteristics were only available for INSPIRE, we restricted this analysis to participants enrolled in this cohort. We evaluated associations between the above-mentioned parameters and newborn metabolite concentrations. Prenatal stress exposure was ascertained from questionnaires administered at the infant one-year visit. An affirmative response to experiencing separation/divorce, death of a loved one, high stress job, financial troubles, unemployment, partner unemployment, or any other stressor (participant provided) was defined as prenatal stress exposure (categorized as one stressor, multiple stressors, or no stressors in analyses) [15]. SVI was calculated using methods outlined by the Centers for Disease Control/Agency for Toxic Substances and Disease Registry (CDC/ATSDR) [16]. SVI ranged from 0-1, with 0 representing the lowest vulnerability census tracts and 1 representing the highest vulnerability census tracts (i.e., SVI was measured at the census tract level, not participant level). Residence (urban, rural) and type of insurance coverage (private, government, other) were ascertained from questionnaires administered at enrollment. Residence was determined based on zip code and categorized according to the 2010 Census Urban and Rural Classification and Urban Area Criteria [17]. For this secondary analysis, we utilized the same statistical plan carried out in the primary analysis. All maternal health

characteristics (those included in the primary analysis in addition to prenatal stress, SVI, residence, and type of insurance coverage) were included in the MANOVA analyses and in the subsequent separate multivariable linear/proportional odds regression models to reduce multiple testing and account for potential confounding. We calculated point estimates and confidence intervals for an IQR difference increase in SVI. None of the maternal characteristics were highly correlated (**Figure S3**).

To test the hypothesis that maternal health characteristics may increase the risk of later life metabolic dysfunction in offspring, we additionally explored the relationship between metabolites that were significantly associated with maternal health characteristics in the primary and secondary analyses and child BMI from ages 1-3 years. This analysis was performed among subsets of the INSPIRE and Healthy Start cohorts with available weight and height measurements. For this analysis, we pooled INSPIRE and Healthy Start participants to increase power. We performed longitudinal linear mixed-effects regression modeling to assess associations between metabolite concentrations at birth and repeated child BMI measures from ages 1-3 years. In this analysis, we included participant ID as a random effect and adjusted for cohort, whether the recumbent length/standing height was estimated, age in days at BMI measurement, birth weight, gestational age, infant race, infant ethnicity, and sex. P-values for interactions between time (years) and metabolites were calculated using likelihood ratio tests. Continuous metabolites were log-transformed. Data analyses were performed using R software, version 4.2.2 (R Foundation for Statistical Computing, Vienna, Austria).

References

1. National Institutes of Health: Newborn Screening. Available online: https://www.nichd.nih.gov/health/topics/newborn (accessed on 10 June 2022).
2. CLSI. *Newborn Screening by Tandem Mass Spectrometry*. 2nd ed. CLSI guideline NBS04. Wayne, PA: Clinical and Laboratory Standards Institute; 2017.
3. Health Resources & Services Administration: Recommended uniform screening panel. Available online: https://www.hrsa.gov/advisory-committees/heritable-disorders/rusp/index.html (accessed on 10 June 2022).
4. Feuchtbaum, L.; Carter, J.; Dowray, S.; Currier, R.J.; Lorey, F. Birth prevalence of disorders detectable through newborn screening by race/ethnicity. *Genet Med* 2012, 14, 937-945.
5. Donovan, B.M.; Ryckman, K.K.; Breheny, P.J.; Gebretsadik, T.; Turi, K.N.; Larkin, E.K.; Li, Y.; Dorley, M.C.; Hartert, T.V. Association of newborn screening metabolites with risk of wheezing in childhood. *Pediatr Res* 2018, 84, 619-624.
6. de Onis, M.; Onyango, A.; Borghi, E.; Siyam, A.; Blössner, M.; Lutter, C. Worldwide implementation of the WHO Child Growth Standards. *Public Health Nutr* 2012, 15, 1603-1610.
7. Flegal, K.M.; Cole, T.J. Construction of LMS parameters for the Centers for Disease Control and Prevention 2000 growth charts. *Natl Health Stat Report* 2013, 1-3.
8. UpToDate: Measurement of growth in children. Available online: https://www.uptodate.com/contents/measurement-of-growth-in-children?search=calculator-cdc-nchs-infant-weight-for-length-percentiles-less-than&sectionRank=2&usage_type=default&anchor=H3361608930&source=machineLea

rning&selectedTitle=1~150&display_rank=1#H3361608930 (accessed on 11 November 2022).

9. Calculator: WHO infant length for age percentiles (<24 months). Available online: https://www.uptodate.com/contents/calculator-who-infant-length-for-age-percentiles-less-than24-months?search=calculator-cdc-nchs-infant-weight-for-length-percentiles-less-than&topicRef=5356&source=see_link (accessed on 10 December 2022).
10. Calculator: CDC height for age percentiles for females (2 to 20 years). Available online: https://www.uptodate.com/contents/calculator-cdc-height-for-age-percentiles-for-females-2-to-20-years?search=calculator-cdc-nchs-infant-weight-for-length-percentiles-less-than&topicRef=5356&source=see_link (accessed on 10 December 2022).
11. Calculator: CDC height for age percentiles for males (2 to 20 years). Available online: https://www.uptodate.com/contents/calculator-cdc-height-for-age-percentiles-for-males-2-to-20-years?search=calculator-cdc-nchs-infant-weight-for-length-percentiles-less-than&topicRef=5356&source=see_link (accessed on 10 December 2022).
12. van Buuren, S. and K. Groothuis-Oudshoorn. mice: Multivariate Imputation by Chained Equations in R. *J Stat Softw* 2011, 45(3), 1-67.
13. von Hippel, P.T. Regression with Missing Ys: An Improved Strategy for Analyzing Multiply Imputed Data. *Sociol Methodol* 2007, 37, 83-117.
14. Dambrova, M.; Makrecka-Kuka, M.; Kuka, J.; Vilskersts, R.; Nordberg, D.; Attwood, M.M.; Smesny, S.; Sen, Z.D.; Guo, A.C.; Oler, E.; et al. Acylcarnitines: Nomenclature, Biomarkers, Therapeutic Potential, Drug Targets, and Clinical Trials. *Pharmacol Rev* 2022, 74, 506-551.

15. Brunwasser, S.M.; Slavich, G.M.; Newcomb, D.C.; Gebretsadik, T.; Turi, K.N.; Stone, C., Jr.; Anderson, L.J.; Hartert, T.V. Sex-specific association between prenatal life stress exposure and infant pro-inflammatory cytokine levels during acute respiratory infection. *Brain Behav Immun* 2019, 76, 275-279.
16. CDC SVI Documentation 2020. Available online: https://www.atsdr.cdc.gov/placeandhealth/svi/documentation/SVI_documentation_2020.html (accessed on 7 December 2022).
17. 2010 census urban and rural classification and urban area criteria. Available online: https://www.census.gov/programs-surveys/geography/guidance/geo-areas/urban-rural/2010-urban-rural.html (accessed on 4 December 2022).

Supplementary figure legends

Figure S1. Newborn screening metabolite distributions in the discovery cohorts (INSPIRE: n=1920, MARCH: n=365).

Figure S2. Correlation between maternal health characteristics in the discovery cohorts (n=2285).

Figure S3. Correlation between prenatal stress exposure, Social Vulnerability Index, residence, and type of insurance and all other maternal health characteristics in INSPIRE (n=1920).

Figure S4. Higher pre-pregnancy BMI and higher age at delivery are associated with increased free carnitine (C0) and acetylcarnitine (C2) at birth, respectively, in both the discovery (n=2264) and replication (n=1201) cohorts after excluding women with potentially implausible pre-pregnancy BMIs (>50). Multivariable linear regression was used to assess the association between higher BMI and C0 and higher age at delivery and C2. These analyses were adjusted for birth weight, gestational age, infant race, infant ethnicity, sex, cohort (discovery phase only), and all other maternal health characteristics. C0 and C2 were log-transformed. Point estimates were estimated for an 8.48 unit (interquartile range) increase in pre-pregnancy BMI and an 8-year increase in maternal age at delivery.

Figure S5. Social Vulnerability Index (SVI) is associated with long-chain acylcarnitine and amino acid concentrations at birth, type of insurance coverage is associated with medium- and long-chain acylcarnitine concentrations at birth, and residence is associated with medium-chain acylcarnitine concentrations at birth in INSPIRE (n=1920). Multivariate analysis of variance was used to assess the associations between prenatal stress exposure, SVI, type of insurance, and residence and established metabolite groups. This analysis was adjusted for birth weight, gestational age, infant race, infant ethnicity, sex, and all other maternal health characteristics (those included in the primary analysis [prenatal smoking, pre-pregnancy BMI, education, occupational status, marital status, age at delivery, asthma, gestational diabetes, and mode of delivery], in addition to prenatal stress, SVI, residence, and type of insurance coverage). The horizontal line indicates p=0.05.

Figure S6. Statistically significant associations between Social Vulnerability Index, type of insurance coverage, and residence and newborn metabolite concentrations in INSPIRE (n=1920). Multivariable linear regression was used to assess the associations between Social Vulnerability Index, type of insurance coverage, and residence and newborn metabolite concentrations. These analyses were adjusted for birth weight, gestational age, infant race, infant ethnicity, and sex, and all other maternal health characteristics. Metabolites were log-transformed. Point estimates were estimated for a 0.51 unit (interquartile range) increase in Social Vulnerability Index.

Figure S7. Distribution of child BMI measurements at ages 1, 2, and 3 years in INSPIRE (n=1692) and Healthy Start (n=1143).
