# Supplementary material for: The Associations of Maternal Health Characteristics, Newborn Metabolite Concentrations, and Child Body Mass Index among US Children in the ECHO Program
Source: Metabolites. 2023 Apr 1;13(4):510. doi: 10.3390/metabo13040510 (PMC10144800; doi:10.3390/metabo13040510)
Supplement: Supplementary file 1 [file metabolites-13-00510-s001.zip › Table S1.pdf]

Table S1. Newborn screening (NBS) metabolites measured in each cohort.

Metabolite Name (units)	Metabolite Abbreviation	Measurement collected on NBS panel by cohort		
		INSPIRE	MARCH	Healthy Start
Free carnitine (umol/L)	C0	X	X	X
**Short-chain acylcarnitines (umol/L)**				
Acetylcarnitine	C2	X	X	X
Propionylcarnitine	C3	X	X	X
Butyrylcarnitine + Isobutyrylcarnitine	C4	X	X	X
Isovalerylcarnitine + Methylbutyrylcarnitine	C5	X	X	X
Tiglylcarnitine	C5:1	X	X	X
3-Hydroxyisovalerylcarnitine	C5-OH		X	
Glutarylcarnitine	C5-DC		X	
**Medium-chain acylcarnitines (umol/L)**				
Hexanoylcarnitine	C6	X	X	X
Methylglutarylcarnitine	C6-DC	X		X
Octanoylcarnitine	C8	X	X	X
Octenoylcarnitine	C8:1		X	X
Decanoylcarnitine	C10	X	X	X

Decenoylcarnitine	C10:1	X	X	X
Decadienoylcarnitine	C10:2	X	X	X
Dodecanoylcarnitine	C12		X	X
Dodecenoylcarnitine	C12:1		X	X
**Long-chain acylcarnitines (umol/L)**				
Tetradecanoylcarnitine	C14	X	X	X
3-Hydroxytetradecanoylcarnitine	C14-OH	X	X	X
Tetradecenoylcarnitine	C14:1	X	X	X
Tetradecadienoylcarnitine	C14:2		X	X
Palmitoylcarnitine	C16	X	X	X
3-Hydroxypalmitoylcarnitine	C16-OH	X	X	X
Palmitoleylcarnitine	C16:1	X	X	X
3-Hydroxypalmitoleylcarnitine	C16:1-OH			X
Heptadecanoylcarnitine	C17		X	
Stearoylcarnitine	C18	X	X	X
3-Hydroxystearoylcarnitine	C18-OH		X	X
Oleoylcarnitine	C18:1	X	X	X
3-Hydroxyoleoylcarnitine	C18:1-OH	X	X	X
Linoleoylcarnitine	C18:2	X	X	X
**Amino Acids (umol/L)**				
Alanine	ALA			X
Arginine	ARG	X	X	X
Argininosuccinate	ASA	X	X	

Citrulline	CIT	X	X	X
Glycine	GLY	X	X	X
Leucine	LEU	X	X	
Methionine	MET	X	X	X
Ornithine	ORN	X	X	X
Phenylalanine	PHE	X	X	X
Proline	PRO		X	
Succinylacetone	SUAC	X		
Tyrosine	TYR	X	X	X
Valine	VAL	X	X	X
**Total**		**33**	**40**	**37**
