# Supplementary material for: The Associations of Maternal Health Characteristics, Newborn Metabolite Concentrations, and Child Body Mass Index among US Children in the ECHO Program
Source: Metabolites. 2023 Apr 1;13(4):510. doi: 10.3390/metabo13040510 (PMC10144800; doi:10.3390/metabo13040510)
Supplement: Supplementary file 1 [file metabolites-13-00510-s001.zip › Table S2.pdf]

Table S2. Ascertainment of maternal health characteristics by cohort.

Maternal health characteristic	Cohort		
	**INSPIRE**	**MARCH**	**Healthy Start**
Prenatal smoking	a	b	b
Pre-pregnancy BMI	a	b	b
Education	a	b	b
Occupational status	a	b	b
Marital status	a	b	b
Age at delivery	a	d	c
Asthma	a	b	b
Gestational diabetes	a	d	c
Mode of delivery	a	d	c

a: Ascertained from questionnaires administered at enrollment (infant age ~2 months).

b: Ascertained from questionnaires administered during pregnancy.

c: Ascertained from medical record at delivery.

d: Ascertained from birth certificates.
