# Supplementary material for: The Associations of Maternal Health Characteristics, Newborn Metabolite Concentrations, and Child Body Mass Index among US Children in the ECHO Program
Source: Metabolites. 2023 Apr 1;13(4):510. doi: 10.3390/metabo13040510 (PMC10144800; doi:10.3390/metabo13040510)
Supplement: Supplementary file 1 [file metabolites-13-00510-s001.zip › Table S3.pdf]

Table S3. Newborn screening metabolite concentrations (n=31) by cohort.

Metabolite (umol/L)	Cohort			
	INSPIRE	MARCH	Healthy Start	p-value[a]
**Total sample size**	**1920**	**365**	**1207**	
Free carnitine (C0), median (IQR)	18.13 (14.11-23.30)	20.07 (14.75-25.04)	30.50 (24.00-37.70)	<0.001*
Missing, N (%)	7 (0)	0 (0)	1 (0)	
**Acylcarnitines**				
Acetylcarnitine (C2), median (IQR)	21.17 (16.91-27.00)	21.58 (17.15-27.44)	25.50 (21.70-30.40)	<0.001*
Missing, N (%)	7 (0)	0 (0)	1 (0)	
Propionylcarnitine (C3), median (IQR)	1.43 (1.09-1.85)	2.19 (1.66-2.85)	1.72 (1.31-2.16)	<0.001*
Missing, N (%)	7 (0)	0 (0)	1 (0)	
Butyrylcarnitine + Isobutyrylcarnitine (C4), median (IQR)	0.21 (0.17-0.26)	0.21 (0.16-0.29)	0.27 (0.20-0.35)	<0.001*
Missing, N (%)	7 (0)	0 (0)	1 (0)	
Isovalerylcarnitine + Methylbutyrylcarnitine (C5), median (IQR)	0.08 (0.07-0.11)	0.09 (0.07-0.12)	0.12 (0.09-0.16)	<0.001*
Missing, N (%)	12 (1)	0 (0)	1 (0)	
Tiglylcarnitine (C5:1), N (%)				<0.001*
0 - <0.01	203 (11)	72 (20)	125 (10)	

≥0.01 - <0.02	1696 (88)	286 (78)	144 (12)	
≥0.02 - <0.03	13 (1)	7 (2)	110 (9)	
≥0.03	1 (0)	0 (0)	827 (69)	
Missing, N (%)	7 (0)	0 (0)	1 (0)	
Hexanoylcarnitine (C6), median (IQR)	0.04 (0.04-0.06)	0.05 (0.04-0.06)	0.07 (0.06-0.09)	<0.001*
Missing, N (%)	7 (0)	0 (0)	1 (0)	
Octanoylcarnitine (C8), median (IQR)	0.06 (0.05-0.07)	0.05 (0.04-0.06)	0.08 (0.06-0.11)	<0.001*
Missing, N (%)	7 (0)	0 (0)	1 (0)	
Decanoylcarnitine (C10), median (IQR)	0.08 (0.06-0.11)	0.08 (0.06-0.11)	0.13 (0.10-0.17)	<0.001*
Missing, N (%)	7 (0)	0 (0)	1 (0)	
Decenoylcarnitine (C10:1), median (IQR)	0.05 (0.04-0.06)	0.04 (0.03-0.05)	0.07 (0.05-0.10)	<0.001*
Missing, N (%)	7 (0)	0 (0)	1 (0)	
Decadienoylcarnitine (C10:2), N (%)				<0.001*
0 - <0.01	48 (3)	69 (19)	337 (28)	
≥0.01 - <0.02	1771 (92)	288 (79)	201 (17)	
≥0.02 - <0.03	90 (5)	8 (2)	208 (17)	
≥0.03	4 (0)	0 (0)	460 (38)	
Missing, N (%)	7 (0)	0 (0)	1 (0)	
Tetradecanoylcarnitine (C14), median (IQR)	0.21 (0.17-0.27)	0.22 (0.17-0.27)	0.25 (0.20-0.31)	<0.001*
Missing, N (%)	7 (0)	0 (0)	1 (0)	

3-Hydroxytetradecan-oylcarnitine (C14-OH), N (%)				<0.001*
0 - <0.01	33 (2)	9 (2)	55 (5)	
≥0.01 - <0.02	934 (49)	141 (39)	67 (6)	
≥0.02 - <0.03	723 (38)	153 (42)	151 (13)	
≥0.03 - <0.04	183 (10)	43 (12)	196 (16)	
≥0.04 - <0.05	36 (2)	16 (4)	210 (17)	
≥0.05	4 (0)	3 (1)	527 (44)	
Missing, N (%)	7 (0)	0 (0)	1 (0)	
Tetradecenoylcarnitine (C14:1), median (IQR)	0.12 (0.09-0.17)	0.12 (0.08-0.16)	0.15 (0.11-0.19)	<0.001*
Missing, N (%)	7 (0)	0 (0)	1 (0)	
Palmitoylcarnitine (C16), median (IQR)	2.81 (2.25-3.45)	3.56 (2.78-4.19)	2.87 (2.35-3.43)	<0.001*
Missing, N (%)	7 (0)	0 (0)	1 (0)	
3-Hydroxypalmitoylca-rnitine (C16-OH), N (%)				<0.001*
0 - <0.01	4 (0)	1 (0)	51 (4)	
≥0.01 - <0.02	390 (20)	51 (14)	47 (4)	
≥0.02 - <0.03	967 (50)	139 (38)	194 (16)	
≥0.03 - <0.04	418 (22)	117 (32)	260 (22)	
≥0.04 - <0.05	106 (6)	43 (12)	231 (19)	
≥0.05	28 (1)	14 (4)	423 (35)	
Missing, N (%)	7 (0)	0 (0)	1 (0)	

Palmitoleylcarnitine (C16:1), median (IQR)	0.21 (0.16-0.27)	0.24 (0.19-0.30)	0.21 (0.17-0.27)	<0.001*
Missing, N (%)	7 (0)	0 (0)	1 (0)	
Stearoylcarnitine (C18), median (IQR)	0.77 (0.61-0.95)	0.86 (0.72-1.03)	0.84 (0.68-1.03)	<0.001*
Missing, N (%)	7 (0)	0 (0)	1 (0)	
Oleoylcarnitine (C18:1), median (IQR)	1.16 (0.93-1.42)	1.22 (0.99-1.44)	1.05 (0.87-1.27)	<0.001*
Missing, N (%)	7 (0)	0 (0)	1 (0)	
3-Hydroxyoleoylcarni-tine (C18:1-OH), N (%)				<0.001*
0 - <0.01	9 (0)	0 (0)	141 (12)	
≥0.01 - <0.02	529 (28)	83 (23)	97 (8)	
≥0.02 - <0.03	1101 (57)	212 (58)	304 (25)	
≥0.03 - <0.04	251 (13)	64 (18)	246 (20)	
≥0.04	23 (1)	6 (2)	418 (35)	
Missing, N (%)	7 (0)	0 (0)	1 (0)	
Linoleoylcarnitine (C18:2), median (IQR)	0.16 (0.12-0.22)	0.19 (0.15-0.25)	0.16 (0.12-0.21)	<0.001*
Missing, N (%)	7 (0)	0 (0)	1 (0)	
**Amino Acids**				
Arginine (ARG), median (IQR)	8.00 (6.00-11.00)	8.81 (5.66-12.86)	12.00 (9.00-16.00)	<0.001*
Missing, N (%)	5 (0)	0 (0)	1 (0)	
Argininosuccinate (ASA), median (IQR)	0.06 (0.05-0.08)	0.15 (0.11-0.21)	--	<0.001*

Missing, N (%)	5 (0)	0 (0)	1207 (100)	
Citrulline (CIT), median (IQR)	12.00 (10.00-15.00)	12.80 (10.46-15.51)	13.00 (11.00-16.00)	<0.001*
Missing, N (%)	5 (0)	0 (0)	1 (0)	
Glycine (GLY), median (IQR)	383.00 (329.00-443.00)	518.34 (452.84-595.28)	480.00 (407.00-564.00)	<0.001*
Missing, N (%)	5 (0)	0 (0)	1 (0)	
Leucine (LEU), median (IQR)	89.00 (77.00-104.00)	84.70 (71.01-100.49)	--	<0.001*
Missing, N (%)	5 (0)	0 (0)	1207 (100)	
Methionine (MET), median (IQR)	19.00 (16.00-22.00)	22.54 (18.52-27.56)	25.00 (21.00-30.00)	<0.001*
Missing, N (%)	5 (0)	0 (0)	1 (0)	
Ornithine (ORN), median (IQR)	66.00 (55.00-81.00)	64.20 (53.20-79.51)	75.00 (65.00-88.00)	<0.001*
Missing, N (%)	5 (0)	0 (0)	1 (0)	
Phenylalanine (PHE), median (IQR)	49.00 (43.00-55.00)	50.22 (43.57-59.22)	53.00 (47.00-60.00)	<0.001*
Missing, N (%)	7 (0)	0 (0)	1 (0)	
Tyrosine (TYR), median (IQR)	78.00 (61.00-99.00)	77.20 (62.90-97.33)	79.00 (63.25-101.00)	0.21
Missing, N (%)	7 (0)	0 (0)	1 (0)	
Valine (VAL), median (IQR)	85.00 (73.00-101.00)	89.28 (76.03-103.77)	103.00 (89.00-124.00)	<0.001*
Missing, N (%)	7 (0)	0 (0)	1 (0)	

IQR, interquartile range.

*P<0.05

[a]P-values for comparisons between cohorts calculated using Kruskal-Wallis or Pearson χ^2 test, as appropriate.

-- Metabolite not collected.
