# Supplementary material for: The Associations of Maternal Health Characteristics, Newborn Metabolite Concentrations, and Child Body Mass Index among US Children in the ECHO Program
Source: Metabolites. 2023 Apr 1;13(4):510. doi: 10.3390/metabo13040510 (PMC10144800; doi:10.3390/metabo13040510)
Supplement: Supplementary file 1 [file metabolites-13-00510-s001.zip › Table S4.pdf]

Table S4. **Pre-specified metabolite groups included in stage one of the statistical plan.**

Metabolite group	Metabolite name
Short-chain acylcarnitines	Acetylcarnitine (C2)
	Propionylcarnitine (C3)
	Butyrylcarnitine + Isobutyrylcarnitine (C4)
	Isovalerylcarnitine + Methylbutyrylcarnitine (C5)
Medium-chain acylcarnitines	Hexanoylcarnitine (C6)
	Octanoylcarnitine (C8)
	Decanoylcarnitine (C10)
	Decenoylcarnitine (C10:1)
Long-chain acylcarnitines	Tetradecanoylcarnitine (C14)
	Tetradecenoylcarnitine (C14:1)
	Palmitoylcarnitine (C16)
	Palmitoylcarnitine (C16:1)
	Stearoylcarnitine (C18)
	Oleoylcarnitine (C18:1)
	Linoleoylcarnitine (C18:2)
Amino acids	Arginine (ARG)
	Argininosuccinate (ASA)[a]
	Citrulline (CIT)
	Glycine (GLY)
	Leucine (LEU)[a]
	Methionine (MET)

	Ornithine (ORN)
	Phenylalanine (PHE)
	Tyrosine (TYR)
	Valine (VAL)

[a]Metabolite was not collected in replication cohort and, therefore, not included in replication analyses.
