# Supplementary material for: The Associations of Maternal Health Characteristics, Newborn Metabolite Concentrations, and Child Body Mass Index among US Children in the ECHO Program
Source: Metabolites. 2023 Apr 1;13(4):510. doi: 10.3390/metabo13040510 (PMC10144800; doi:10.3390/metabo13040510)
Supplement: Supplementary file 1 [file metabolites-13-00510-s001.zip › Table S5.pdf]

Table S5. **Comparison of characteristics for MARCH participants with newborn screening (NBS) metabolic data and all MARCH participants.**

	MARCH participants with NBS data	All MARCH participants	p-value[a]
**Sample size**	**365**	**947**	
**Maternal characteristics**			
Prenatal smoking, N (%)	41 (11)	104 (11)	1
Missing, N (%)	47 (13)	129 (14)	
Pre-pregnancy BMI, mean (SD)	29 (8)	29 (8)	0.29
Missing, N (%)	43 (12)	123 (13)	
Education, N (%)			0.60
<High school	31 (8)	82 (9)	
High school degree	68 (19)	173 (18)	
Some college	121 (33)	279 (29)	
≥College degree	100 (27)	286 (30)	
Missing, N (%)	45 (12)	127 (13)	
Occupational status, N (%)			0.94
Not employed	81 (22)	211 (22)	
Employed	239 (65)	609 (64)	

Missing, N (%)	45 (12)	127 (13)	
Marital status, N (%)			0.42
Not married	177 (48)	431 (46)	
Married	142 (39)	388 (41)	
Missing, N (%)	46 (13)	128 (14)	
Age at delivery (years), mean (SD)	29 (6)	29 (6)	0.74
Missing, N (%)	71 (19)	99 (10)	
Asthma, N (%)	67 (18)	160 (17)	0.64
Missing, N (%)	46 (13)	128 (14)	
Gestational diabetes, N (%)	23 (6)	58 (6)	0.67
Missing, N (%)	71 (19)	100 (11)	
C-section, N (%)	105 (29)	313 (33)	0.77
Missing, N (%)	71 (19)	99 (10)	
**Infant characteristics**			
Birth weight (grams), mean (SD)	3225 (577)	3204 (618)	0.73
Missing, N (%)	71 (19)	99 (10)	
Gestational age (weeks), mean (SD)	38 (2)	38 (2)	0.86
Missing, N (%)	71 (19)	99 (10)	
Infant race, N (%)			0.27

White	195 (53)	497 (52)	
Black	94 (26)	264 (28)	
Other	46 (13)	91 (10)	
Missing, N (%)	30 (8)	95 (10)	
Hispanic ethnicity, N (%)	34 (9)	58 (10)	0.07
Missing, N (%)	30 (8)	95 (10)	
Male sex, N (%)	137 (38)	415 (44)	0.53
Missing, N (%)	71 (19)	99 (10)	

SD, standard deviation.

*P<0.05

[a]P-value for comparisons between cohorts calculated using Kruskal-Wallis or Pearson χ^2 test, as appropriate.
