# Supplementary material for: The Associations of Maternal Health Characteristics, Newborn Metabolite Concentrations, and Child Body Mass Index among US Children in the ECHO Program
Source: Metabolites. 2023 Apr 1;13(4):510. doi: 10.3390/metabo13040510 (PMC10144800; doi:10.3390/metabo13040510)
Supplement: Supplementary file 1 [file metabolites-13-00510-s001.zip › Table S7.pdf]

Table S7. Additional maternal health characteristics of INSPIRE participants with linked newborn screening metabolic data (n=1920).

Characteristic	
Prenatal stress exposure, N (%)	
No stressors	1168 (61)
One stressor	389 (20)
Multiple stressors	183 (10)
Missing, N (%)	180 (9)
Social Vulnerability Index, mean (SD)	0.51 (0.29)
Missing, N (%)	1 (0)
Residence, N (%)	
Urban	1450 (76)
Rural	453 (24)
Missing, N (%)	17 (1)
Type of insurance coverage, N (%)	
Government	1042 (54)
Private	856 (45)
Other	22 (1)
Missing, N (%)	0

SD, standard deviation.
