# Supplementary figures and images for: The Associations of Maternal Health Characteristics, Newborn Metabolite Concentrations, and Child Body Mass Index among US Children in the ECHO Program
Source: Metabolites. 2023 Apr 1;13(4):510. doi: 10.3390/metabo13040510 (PMC10144800; doi:10.3390/metabo13040510)

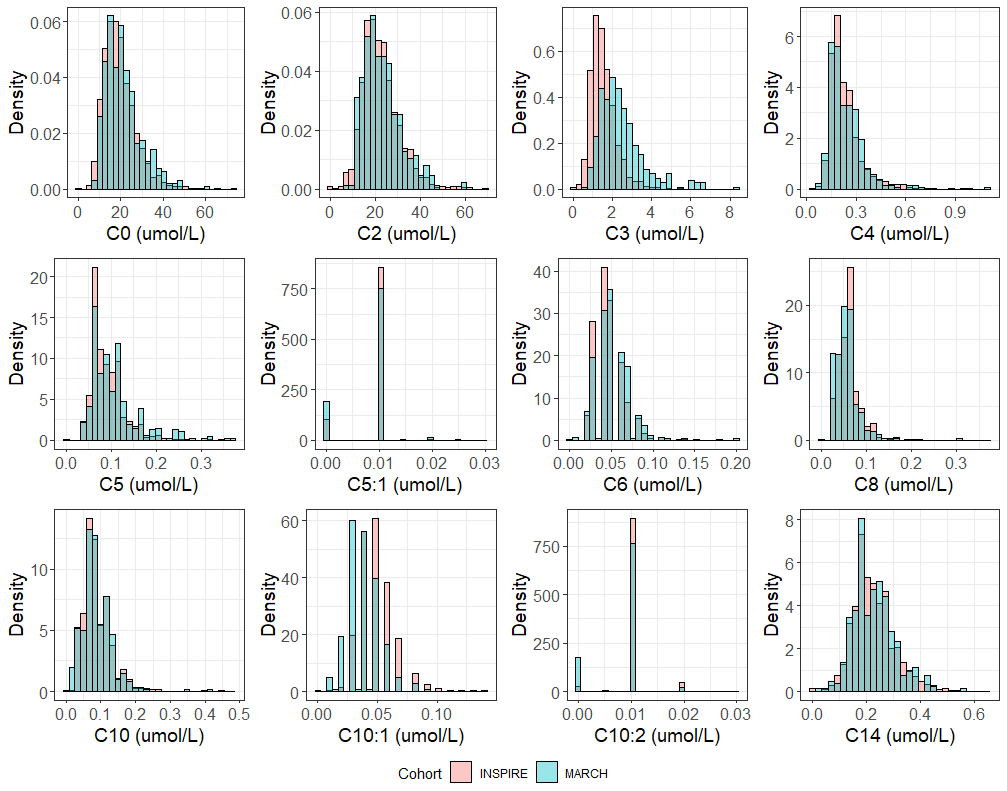

Supplement: Supplementary file 1 [file metabolites-13-00510-s001.zip › Figure_S1_panel_A.png]

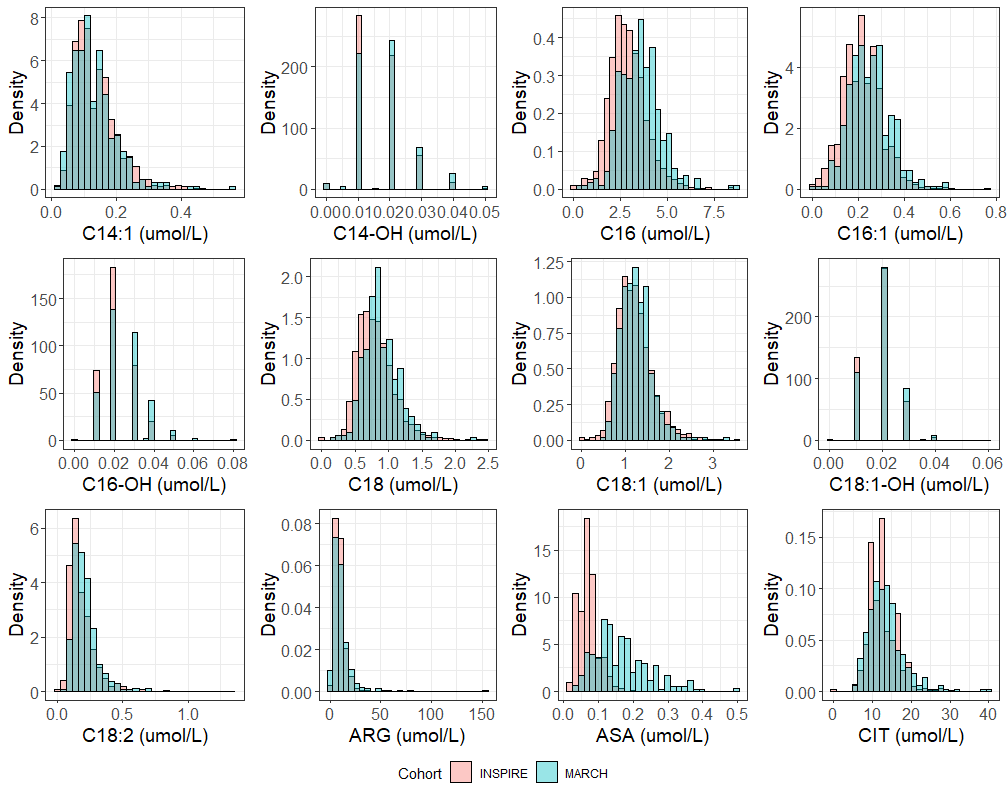

Supplement: Supplementary file 1 [file metabolites-13-00510-s001.zip › Figure_S1_panel_B.png]

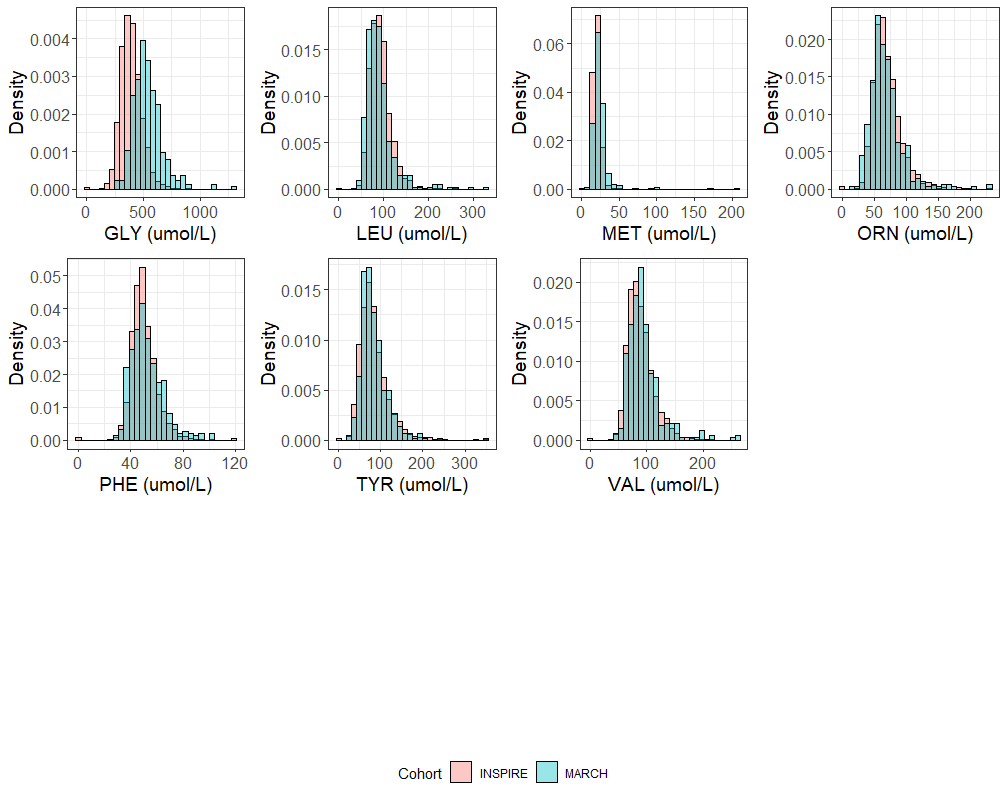

Supplement: Supplementary file 1 [file metabolites-13-00510-s001.zip › Figure_S1_panel_C.png]

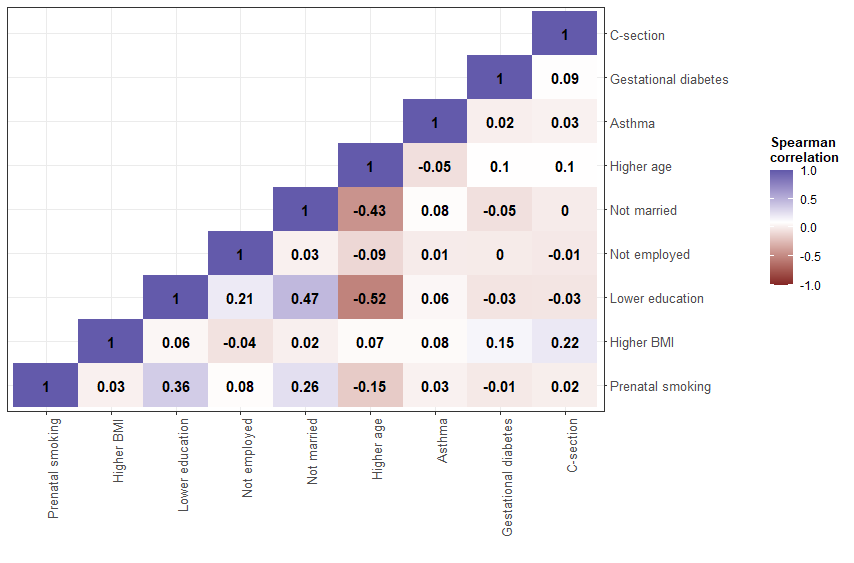

Supplement: Supplementary file 1 [file metabolites-13-00510-s001.zip › Figure_S2.png]

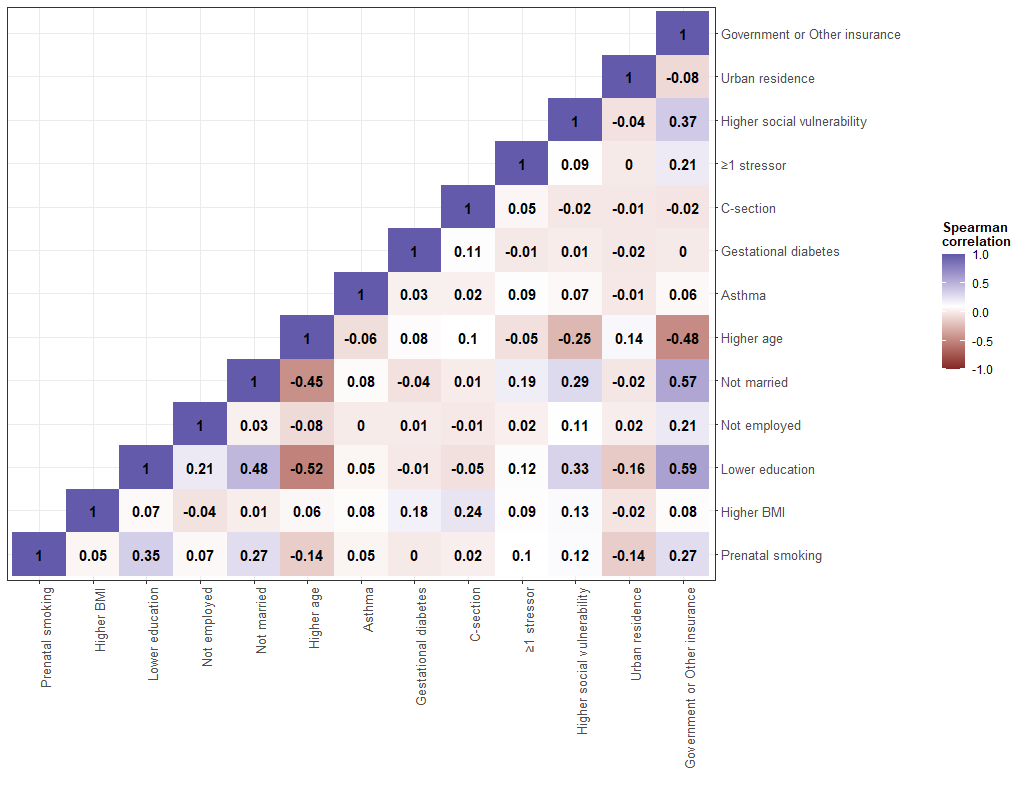

Supplement: Supplementary file 1 [file metabolites-13-00510-s001.zip › Figure_S3.png]

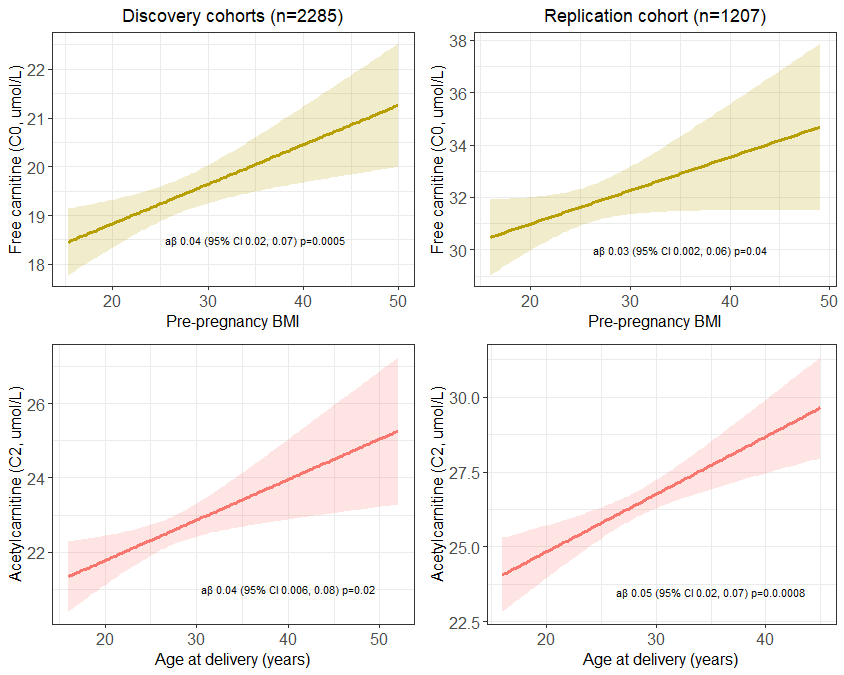

Supplement: Supplementary file 1 [file metabolites-13-00510-s001.zip › Figure_S4.png]

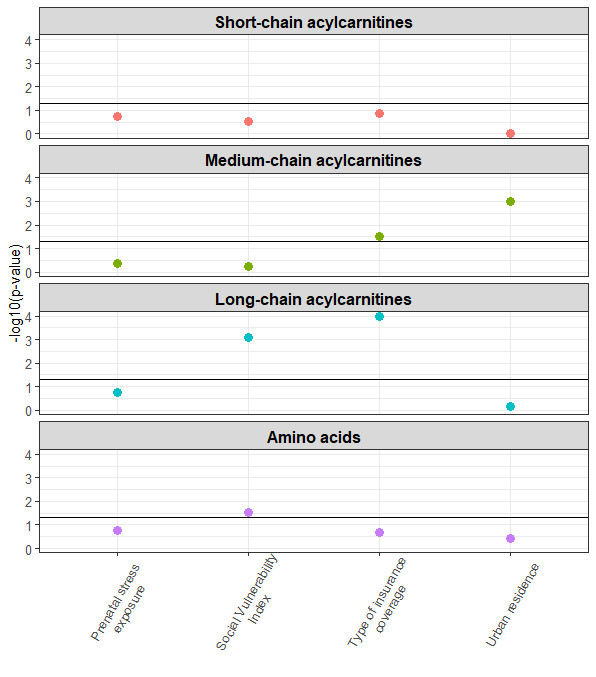

Supplement: Supplementary file 1 [file metabolites-13-00510-s001.zip › Figure_S5.png]

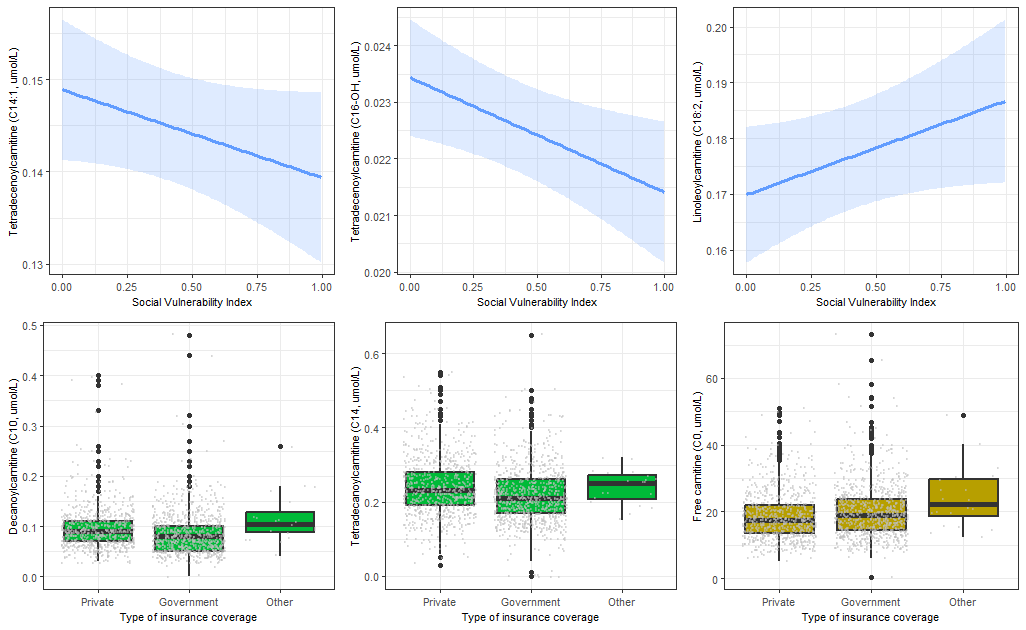

Supplement: Supplementary file 1 [file metabolites-13-00510-s001.zip › Figure_S6_panel_A.png]

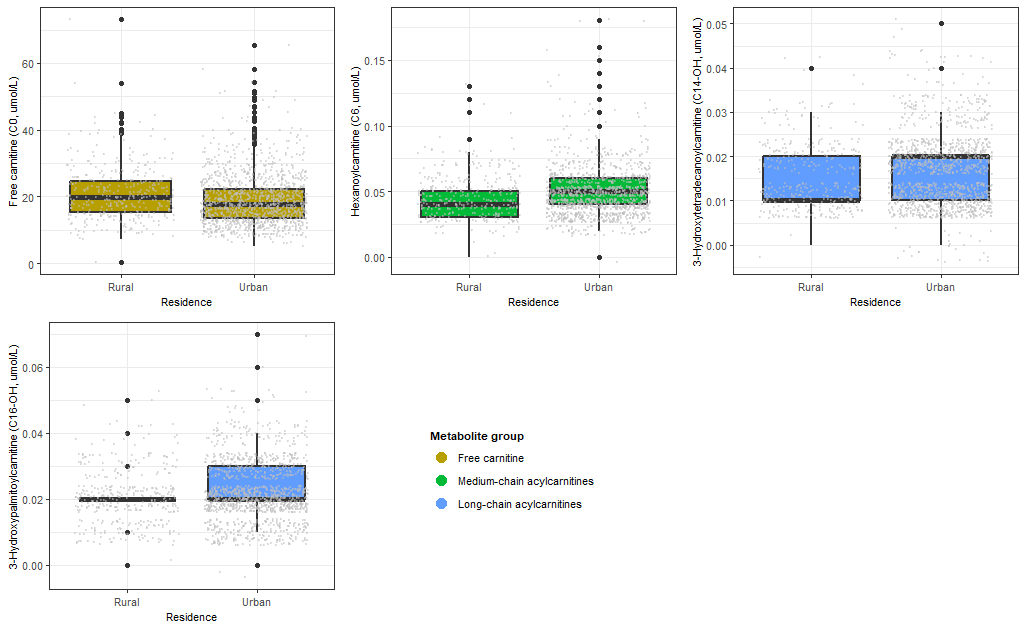

Supplement: Supplementary file 1 [file metabolites-13-00510-s001.zip › Figure_S6_panel_B.png]

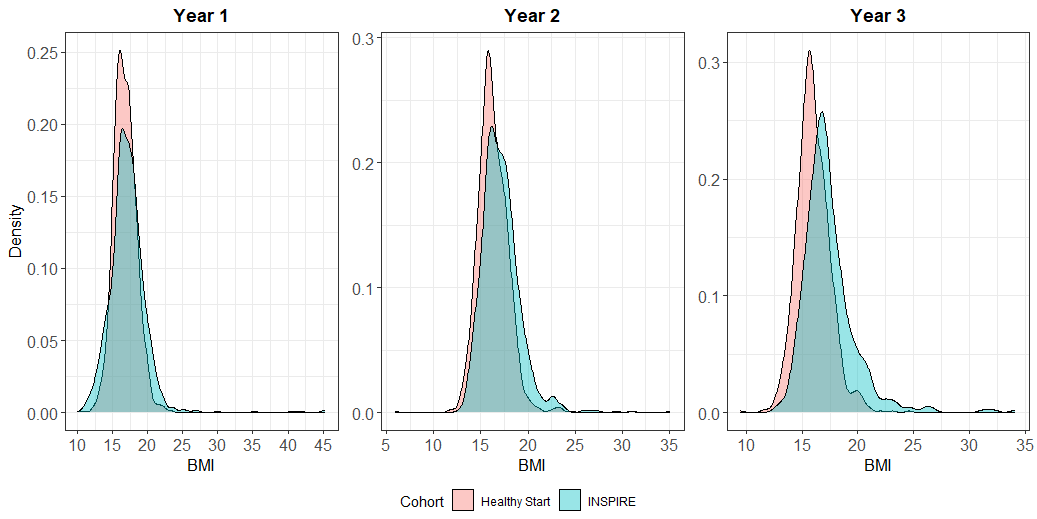

Supplement: Supplementary file 1 [file metabolites-13-00510-s001.zip › Figure_S7.png]
